# Towards an Explanation of the Social Value of Health Systems: An Interpretive Synthesis

**DOI:** 10.34172/ijhpm.2020.159

**Published:** 2020-08-26

**Authors:** Eleanor Beth Whyle, Jill Olivier

**Affiliations:** Health Policy and Systems Division, School of Public Health and Family Medicine, Faculty of Health Sciences, University of Cape Town, Cape Town, South Africa.

**Keywords:** Social Values, Interpretive Synthesis, Health Systems, Complexity, Emergence

## Abstract

**Background:** Health systems are complex social systems, and values constitute a central dimension of their complexity. Values are commonly understood as key drivers of health system change, operating across all health systems components and functions. Moreover, health systems are understood to influence and generate social values, presenting an opportunity to harness health systems to build stronger, more cohesive societies. However, there is little investigation (theoretical, conceptual, or empirical) on social values in health policy and systems research (HPSR), particularly regarding the capacity of health systems to influence and generate social values. This study develops an explanatory theory for the ‘social value of health systems.’

**Methods:** We present the results of an interpretive synthesis of HPSR literature on social values, drawing on a qualitative systematic review, focusing on claims about the relationship between ‘health systems’ and ‘social values.’ We combined relational claims extracted from the literature under a common framework in order to generate new explanatory theory.

**Results:** We identify four mechanisms by which health systems are considered to contribute social value to society: Health systems can: (1) offer a unifying national ideal and build social cohesion, (2) influence and legitimise popular attitudes about rights and entitlements with regard to healthcare and inform citizen’s understanding of state responsibilities, (3) strengthen trust in the state and legitimise state authority, and (4) communicate the extent to which the state values various population groups.

**Conclusion:** We conclude that, using a systems-thinking and complex adaptive systems perspective, the above mechanisms can be explained as emergent properties of the dynamic network of values-based connections operating within health systems. We also demonstrate that this theory accounts for how HPSR authors write about the relationship between health systems and social values. Finally, we offer lessons for researchers and policy-makers seeking to bring about values-based change in health systems.

## Introduction

*“A just system must…be arranged so as to bring about in its members the corresponding sense of justice, and effective desire to act in accordance with its rules for reasons of justice…[Institutions] must be not only just, but framed so as to encourage the virtue of justice in those who take part in them”*—Rawls 1971.^1^

Health systems are complex social systems, and social values constitute a central facet of their complexity.^2-6^ The influence of social values is evident across a myriad of elements, functions and interactions of the health system.

In an earlier systematic review on values in health systems, we found evidence of the influence of values across all health system components and functions.^7^ For example, in service delivery, values are shown to influence preferences for private provision over public^8^ and affect patient-provider relationships,^9^ while with respect to human resources, values impact health provider motivation^10^ and levels of absenteeism.^11^ Within health system governance, values influence the functioning of community accountability mechanisms^12^ and decision-making processes,^13^ and determine macro-level financing arrangements such as the extent of cross-subsidisation.^14^ Values considerations are also increasingly incorporated into technical decision-making processes around health technology assessment.^15^ Critically, across all health system components, values inform the behaviour and choices of individual actors,^16,17^ and shape relationships between actors.^12,18^

The sub-field of health policy analysis has produced substantial evidence suggesting that values influence policy-makers and shape policy-making processes,^19-23^ and, as a result, inform the language of policy documents *and* policy goals.^24-26^ Through this influence on policies, values shape the trajectory of health system development.^27,28^

The earlier review also revealed that values were often positioned by Health Policy and Systems Research (HPSR) authors not only as an *input* influencing health system change, but also as a *property* of health systems. For example, Saltman and Bergman argue that social values determine the existing architecture of health systems and then “continue to influence proposed reforms to that structure,”^29^ while Cleary, Molyneux and Gilson suggest that resource flows reflect the values of a health system.^30^ Others observe that the design of health systems evidence the prevailing values of that society—for example when Kruk et al state that “the design of a health system…conveys important social and political values,”^31^ or van Olmen and colleagues’ suggestion that the prevailing social values “emanate” from the health system.^32^ Values are also described as an *output* of health systems. For example, Gilson states that “a trusting and trusted health system can contribute to building wider social value and social order,”^5^ and Abelson et al argue that health systems contribute to the construction of social values in society.^33^ In the same vein, Frenk notes that it is possible for the state to legitimise certain ideologies through the provision of health services.^34^ These ideas suggest a common understanding that not only are health systems *influenced by* social values, but that, as indicated by Rawls in the quote above, they also have the capacity to *influence* and *generate* social values in the societies they serve.

If this is the case, it is important to improve our understanding of the mechanisms underlying this phenomenon, and whether they can be harnessed to bring about positive social change.

This paper presents an interpretive synthesis of claims about the relationship between social values and health systems in HPSR literature, exploring conceptualisations of the social value of health systems, and developing an initial explanatory theory for the capacity of health systems to generate social value(s). The analysis adapts the steps of Noblit and Hare’s meta-ethnography approach^[1]^, ^35^ and proceeds by synthesising the claims about the relationship between health systems and social values (extracted from the literature) within a unifying frame, and presenting an explanatory theory on the basis of that overarching frame. The explanatory theory draws on foundational HPSR concepts such as emergence and complex causality to lay the conceptual foundations for an explanation of how social values influence, and are influenced and generated by health systems. Finally, we consider the implications of this explanatory theory for researchers and policy-makers—drawing out key lessons for those seeking to understand or contribute to values-based system reform in complex social systems.

## Methods

This interpretive synthesis follows from a prior qualitative systematic review (reported elsewhere) and utilises that collection of evidence^[2]^.^7^ The systematic review applied an iterative approach, based on Boell and Cecez-Kecmanovic’s hermeneutic review methodology.^36^ This allowed for the gradual accumulation of relevant evidence, in accordance with the researchers’ emergent understanding of the key concepts.^37^ The review was limited to peer-reviewed content, including organisational reports, empirical and non-empirical literature, published in English between 1989 and 2018. Two-hundred and eight items were included. Inclusion depended on appearance of the term ‘values’ (or a related term) with a collective modifier (such as ‘national,’ ‘political,’ or ‘community’). This restriction excluded materials using the term ‘values’ only in the numerical sense, or in the sense of ‘importance’ or ‘benefit.’

The systematic review revealed the scope and quantity of HPSR evidence on social values, but concluded that further analysis, allowing for deeper engagement with the evidence, would be beneficial. In particular, we identified multiple relational claims that suggested that health systems can play an important social role in the societies in which they are embedded, and that values are a key determinant of how well systems perform this function. In addition, while the statements about social values in health systems identified in the primary material made a variety of different claims, the claims were not necessarily contradictory, but could be interpreted as complimentary—in other words as telling different parts of a single story. We therefore concluded that a further investigation utilising an interpretive approach, and synthesising the full diversity of claims, would be important for further theoretical development, which was clearly lacking in the existing literature. To this end, we re-reviewed the included papers, excluding those in which the nature of the relationship between social values and health systems was not clear (19 papers in total), and extracting further detailed information on how the relationship between health systems and social values was presented in each paper.

Data extraction was conducted by the first author (EBW). Papers were read and claims about values in relation to health systems identified. These claims were extracted verbatim and then simplified. The extraction and simplification steps were then checked by the second author (JO). Relational claims that were open to interpretation or difficult to simplify were discussed between authors until a consensus interpretation was reached. During analysis, the simplified version of the claim was always viewed concurrently with the verbatim quotes to ensure that nuance was retained in interpretation.

Interpretive synthesis is useful to synthesise qualitative data from a range of qualitative and mixed methods evidence.^37,38^ In contrast to integrative synthesis, which seeks to combine or amalgamate data, interpretive syntheses involves interpretation and induction in order to develop explanatory theory.^39^ Interpretive synthesis seeks to move beyond the collation of primary data and allows for the development of new interpretations at a higher level of abstraction.^40^

We did not extract contextual information about the country or countries of focus in each paper. As such, we were unable to consider in our analysis the impact of particular contextual factors, such as political organisation of the state or level of economic development, on the relationship between health systems and social values. We acknowledge this as a limitation of this synthesis, and hope that the explanatory theory presented here will facilitate future research on the relationship between political and economic contextual factors, health systems and social values.

### Identifying and Categorising Claims About the Relationship Between Health Systems and Social Values

The process of interpretive synthesis began with extracting claims about the relationship between health systems and social values from the evidence-base, and then exploring and categorising the relational claims to identify apparent underlying assumptions and conceptualisations (see data extraction sheet in Supplementary file 1 for the full list of papers and claims extracted).

For the most part, the relational claims presented a simple connection or influence between social values and a particular health system component (such as policies, front-line workers, decision-makers or the health system as a whole), and/or function (such as governance, reform, decision-making or goals) of the health system. Some examples of relational claims, along with the health system component and function they pertain to, are presented in Table.

**Table:** Examples of Relational Claims According to the System Function and Component Referenced

**Relational Claim**	**System Function**	**System Component**
"Social values…form the guiding principles of the healthcare system and currently present a barrier to health priority setting…"^41^ "Much of this [health] priority setting is shaped by the values and perceptions of electorates."^42^	Priority-setting	Health System
"Countries need to customise systems to suit their socio-economic, political and administrative settings. Home-grown health financing systems that resonate with social values will need to be found."^43^ "Health financing arrangements can convey important messages about political priorities and values."^44^	Finance/resource allocation	
"Conflict between the ideology of market-driven health finance and fundamental social and political values proved an even more powerful force for reorienting the competitive reforms than did interest-group opposition."^45^ "Government’s regulatory role is noted to include structuring the system in line with social consensus on the ethical principles...on which it is founded" (Mills and Ranson as quoted in Gilson).^46^	Structure and reform	
"A number of authors highlight the importance of considering societal values and principles as they vary across societies, yet are crucial in determining system goals."^47^	Goals	
"Nurses’ values and worldviews influence their responses to the free care policy."^48^ "Healthcare workers provide care, adhere to guidelines, interact with each other and interact with patients according to their personal values, [and] social and professional norms...among other factors."^49^	Behaviour/decision-making	HCWs and managers
"Values and political ideologies can be central to policy directions through providing a window of opportunity for change, particularly during political electoral cycles."^42^	Agenda	Policy
"The failure of the implementation of these policies, in terms of their equity objectives, can be largely explained by the fact that the absence of equity was never seen as a public issue. Yet for any situation to become a public issue...the question of values is obviously central."^50^	Success/effectiveness/implementation	
"The framing game to be played is dependent on the embedded values of the larger health policy arena...one may expect frames to center on the need to expand social policies to reflect the values inherent in existing programs (and thus, arguably, society)."^51^	Content/structure/framing	
"Policy-makers contested the SMC research evidence mostly due to concerns such as political feasibility, cultural values and discomfort with complex messages."^52^ "Policy actors who prioritised severely ill…argued that the majority of the public would have the same ethical values and expectations for healthcare rationing."^53^	Behaviour/decision-making	Policy-maker/elite
"The cluster of ideas, beliefs, values and attitudes...constitute the normative lens through which policy-makers...interpret and act upon social and political issues."^54^	Perception/expectation	
"Recognizing and aligning policy with ‘values’ underpinning health systems affect whether interventions...are succeeding."^55^ "When the principles of a policy have greater congruency with the social and cultural values within a health system, effective implementation is more likely to occur."^56^	Success/effectiveness/implementation/function	Intervention/program/service
"Policy frames incorporate particular norms of fairness."^57^	Content	
"The incongruences between societal values, institutions and decisions found in Germany may be a central cause behind the significantly lower satisfaction with the system."^58^ "The public’s acceptance of economic evaluation would be limited if the societal values of equity or justice were not incorporated into decision-making."^59^	Perception/expectation	Citizen

Abbreviation: HCWs, healthcare workers.

### Interpreting Relational Claims: The Nature of the Relationship Between Health Systems and Social Values

Before synthesising these relational claims, it was necessary to explore and interpret the nature of the claims to ensure that their meaning and nuance would be retained through the analysis. As mentioned, claims were extracted from both empirical and non-empirical literature. As such, some relational claims presented the product or output of a formal study, while others were given as background knowledge or prescriptive assertions, laying the foundation for the interpretation of data or conceptual development. For the purposes of this study, all relational claims were considered to offer valuable insights into how the relationship is being conceptualised, and were therefore analysed in the same way.

In interpreting the relational claims, three approaches to characterising the nature of the relationship between health systems and social values emerged. Firstly, many of the relational claims used causal language to describe the relationship between health systems and social values, but rarely suggest a simple, or direct causal connection. Secondly, many claims use metaphorical language that implies that health system change is causally dependent on social values (see Box 1). Lastly, we identified a number of relational claims that reverse the direction of influence, suggesting that health systems influence, and even generate, social values. These three types of relational claims will be explored in turn.

**Box 1. Examples of Use of Metaphorical Language to Describe Interactions and Connections**

‘Shape’ metaphors:

“The prevailing settlement underlying a welfare system, however, interacts with, and is shaped by, the changing value base of society.”^5^

“We chose to place it predominantly as a value in this framework since we think values drive and shape the outcomes of health systems.”^60^

“Broader contextual influences seep into the daily practice of a health system through the…values that shape the behaviours of the actors within it.”^16^

‘Drive’ metaphors:

“The values of the community should drive health services.”^61^

“The technology assembly process is not arbitrary, but heavily values driven.”^62^

‘Structural’ metaphors:

“Social and political institutions embodying these norms [truthfulness, solidarity, altruism and fairness] promote affective trust in societies.”^5^

“The dominant institutions underpinning these relationships are not economic incentives and regulatory rules. Instead they are the rules, norms and values that confer responsibilities and rights.”^46^

“The cluster of values surrounding the evolution of the political and social systems sets the scene for the construction of different universal health coverage pathways.”^42^

“Reforms often embody values contrary to those held by health workers.”^63^

‘Mirroring’ metaphors:

“Healthcare services, like other human service systems, mirror the deeply rooted social and cultural expectations of society as a whole.”^64^

“The processes, laws and regulations that define how resources and authority are distributed in the health sector, as well as the volume and type of resources available…are a direct reflection of society›s values.”^64^

“Key dimensions of a country’s healthcare system reflect the core social norms and values held by its citizenry.”^29^

### Causal Language in the Relational Claims

When relational claims make use of causal language they suggest either that social values constitute one influence among many, or posit a causal connection that is dependent on congruence with social values. As an example of the former, Frenk lists ‘ideology’ as one of four forces leading to health system reform, alongside economic, epidemiological and political forces.^34^ Similarly, Renmans et al state that “ideological inclinations and cultural values influence the design of a specific PBF [performance-based financing] scheme.”^65^ In both these cases, social values are understood as a causal factor, operating alongside other causal factors. More explicitly, Saltman and Figueras argue that social values rank, alongside macro-economic factors and demographic issues, as *one of* the most influential contextual factors in health system reform.^64^

In some cases, the relational claims indicate that the influence of social values is conditional—dependent on, or mediated by, alignment or congruence between social values and the health system element in question. For example, Liverani et al list “the framing of evidence in relational to social values” as one of many political and institutional factors influencing the use of evidence in policy-making.^66^ In the same vein, Hanefeld and colleagues’ claim that “recognizing and aligning policy with ‘values’ underpinning health systems [will] affect whether interventions…are succeeding”^55^ suggests a connection between intervention success and the intervention’s degree of alignment with social values.

For these sorts of claims, the interaction in question is often between two health system components (rather than between social values and a health system component), but is dependent on or strengthened by social values. For example, Roberts et al state that “even if [health reformers] lack material resources, [they] can still design political strategies that may give [them] substantial leverage in a policy debate, by wisely using symbols that connect to broad social values.”^67^ This claim posits an interaction between policy-makers and policy outcomes that is contingent on social values.

In addition to claims suggesting that social values constitute one cause among many, and claims suggesting a causal connection that is contingent on social values, many relational claims also position social values as constraining, rather than bringing about, health system change. For example, Redden (writing on the US context) notes that individualistic principles that dominate the current system “preclude consideration” of collective identities and, therefore, of collective rights—entailing that reform efforts come up against the (in)flexibility of “fundamental American values.”^68^ Similarly, Watt et al suggest that implementation can be constrained by “competing management priorities and social norms shaping the interaction between providers and population.”^69^

### Metaphorical Language in the Claims

Many relational claims also use metaphorical language that implies a causal connection. At times, the use of metaphor is explicit and purposeful, such as in Sturmberg and colleagues’ use of the idea of the “healthcare vortex”^27^ as a metaphor for the way in which shared values act as an ‘attractor,’ guiding the behaviour of health system actors, while allowing them to “act in adaptive ways” to generate contextually-specific solutions^[3]^.^70^

Often, however the use of metaphor is less purposeful (and could be unconscious). In these instances, metaphors usually take the place of verbs, and are used to describe how social values interact with health systems (see Box 1 for examples). As is the case with the claims using more literal language, the chosen metaphors often imply, but do not explicitly assert, that the connection in question is causal. However, even on the weakest possible interpretation, the metaphors suggest that a change in social values will result in a change in (some element of) the health system—in other words, that the nature of the health system is, at least partially, a consequence of social values.

### Relational Claims About the Influence of Health Systems on Social Values

In other relational claims, the direction of influence is either reversed (ie, considered as the influence of health systems on social values) or characterised as a mutual influence. For example, Daw et al state that “public support for government programs is partly derived from the design of existing programs that shape public views on who deserves to be a beneficiary, to what extent, and for what services.”^51^ In other words, the design of existing policies shapes users’ ideas about justice and entitlement with regards to healthcare, which in turn influences how users will respond to new policies and programmes.^72^

As noted, a number of these converse relational claims—claims positing the influence of health systems on social values—indicate that the production or promotion of social values is conceived of as a core capacity of health systems. For example, Gilson conceives of health system as “purveyors”^5^ of social values, while Frenk suggests that health systems can “reflect and reinforce” social values, and therefore that health system reform efforts should begin by considering which values the health system should be designed to “promote.”^73^ Similarly, Gilson in 2003 argues that social institutions, such as the health system, can “promote” social values, stating, “social and political institutions embodying these norms [truthfulness, solidarity, and fairness] promote affective trust in societies by committing and enforcing upon all those involved in them a specific set of values.”^5^ Indeed, Sage proposes that health system reform is an *opportunity* to “recalibrate” social values.^74^ These claims suggest that health systems have the capacity to influence social values.

#### Identifying a Line of Argument: The Capacity of the Health System to Influence Social Values

Seeking to better understand how social values could be an *output*of health systems, we explored conceptualisations of the capacity of health systems to influence social values. The relational claims revealed four distinct but related mechanisms, which are explored in turn in this section.

### Health Systems Can Offer a Unifying National Ideal and Build Social Cohesion

Firstly, health systems are frequently conceptualised as symbols of national identity that offer unifying ideals and build social cohesion. Canada presents a particularly striking example: Both Redden,^68^ and Axworthy and Spiegel^75^ argue that the Canadian public healthcare system is an important symbol and defining attribute of national identity. Similarly, Daw et al suggest that Canadians’ strong support for universal health coverage reflects the popular conceptualisation of the health system as a “fundamental cornerstone of Canadian identity”^51^ (see also Giacomini et al^24^). More generally, Kruk et al^31^ and Gilson^5^ propose that, particularly in countries destabilised by violence and conflict, governments can use value-based rehabilitation of health systems to contribute to social cohesion, and create a sense of shared identity.

### Health Systems Can Influence User’s Understanding of Rights, Entitlements and the Appropriate Role of the State in Delivering These

Secondly, health systems are often seen to influence users’ understanding of their rights and entitlements by legitimising ways of working that reflect values. For example Saltman argues that the primary role of the state in the delivery of health services in some Western European countries has been legitimized over time through democratic elections and now constitutes a “deeply rooted norm” in those countries.^64^ Similarly, as noted above, Frenk suggests that the state can use healthcare workers (HCWs) to offer the public “alternatives to magical and religious” worldviews, and can therefore be used to “legitimize different modernising ideologies.”^34^ In this way, health systems can communicate values to the public.^31^

More perniciously, both Kruk et al^31^ and Freedman^76^ argue that user fees and other financial barriers to care legitimate the exclusion of population groups unable to pay. In other words, by systematically denying the poor access to health services, the system can actively shift popular perceptions about rights and entitlements, ultimately legitimising this inequality. This example demonstrates that this legitimizing process is not necessarily a product of users’ direct engagement with the health system, because values can also be legitimised by the “structure of a health system,” as is the case with financial barriers that communicate the acceptability of inequality to users, those excluded, and the broader population.^76^

One of the most clear examples emerging from the literature of this capacity to influence social values and popular norms is the influence of neoliberal economic reforms on the structure of health systems (the health system components that support service delivery, such as financing mechanisms, the role of political oversight, the relationships between them^34,77,78^), and the resultant shift in popular beliefs about the appropriate role of the state in the health system. Beginning in the 1980s, capitalism and neoliberal economic reforms that encouraged market-based mechanisms resulted, in many contexts, in a limiting of the role of the state, for example to the regulation and governance of non-state providers, or to provision only of basic services to the very poor.^8,79^ The balance between state versus market in the provision of healthcare is commonly understood to be an ideological consideration, albeit primarily driven by global trends rather than local values and preferences.^21,77,80^ For example, Reinhardt warns that the incorporation of US-style private health insurance into the Canadian health system will ultimately shift Canada’s “social ethics” to be more like that of the United States.^81^

While the Canadian case reflects a rejection of neoliberal, market-oriented reforms on the basis of values, in other cases neoliberal values have become so deeply embedded as to be considered unchangeable. For example, Heslop and Peterson argue that in the United States, the dominance of market mechanisms for health service delivery has become normalised as a result of the interests of “an organized alliance of health insurance companies and delivery organizations” with an outsized influence on the legislative process, despite the fact that the values implicit in this approach do *not* reflect those of the majority of the US population.^79^ Others considering the US context, however, indicate that social values have been shifted over time as a result of the market-oriented health system structure. Schlesinger for example states that “when goods and services are portrayed as marketable commodities, fairness is defined primarily in terms of individual choice and personal deservingness,”^57^ and Sage agrees that the market values of “public has prized scientific innovation, consumer sovereignty, and personal autonomy” over equity and solidarity.^74^ These examples suggest that health system architecture influences popular social values concerning the appropriate role of the state in health systems.

### Health Systems Can Strengthen Public Trust in the State and Legitimise State Authority

In addition to the capacity of the health system to build a sense of shared identity and values, and influence popular beliefs about health rights and entitlements, a third mechanism by which health systems can contribute value to society is by improving levels of public trust in the state and legitimising state authority.^5,82^ Abelson et al suggest that because “publicly funded health systems comprise such a large degree of state-citizen interaction…mistrust of health systems may contribute to a general mistrust of government.”^33^ In other words, as a site of regular interaction between citizens and the state,^83^ the health system has the capacity to build public trust in the state. This idea is reinforced by Gilson’s suggestion that social institutions (like health systems) that embody social norms can garner public trust, and therefore strengthen the relationship between citizens and the state.^5^ Often, however, this trust is considered contingent on alignment between the values represented by the health system, and dominant social values. Kehoe and Ponting, for example, conducted a study on values as a determinant of trust in health policy-makers, and found that when policies are perceived by the public as misaligned with their values, public trust in government is negatively affected.^84^ Similarly, Abelson et al argue that the trusting relationship between citizens, health professionals and the state that once characterised the UK’s National Health Service (NHS), has been eroded by “consumerism” and “entrepreneurial values.”^85^

This potential of health systems to strengthen the citizen-state relationship by building trust in the state is likely partly a function of users’ direct interaction with the health system, as Abelson et al^33^ and Gilson^86^ suggest. However, other authors argue that accountability mechanisms,^49^ policy decision-making processes,^87,88^ how a health system is financed,^86^ and a history of public action in relation to health systems^86^ all impact the relationship between citizens and the state. This indicates that the architecture of health systems is as important to building value in society as is the direct interaction of patients with health providers. For example, in the UK’s NHS, the system was perceived as ‘fair’ by users as a result of the absence of direct financial incentives affecting the behaviour of providers, which increase user trust in providers.^5^

### Health Systems Can Indicate Extent to Which Various Population Groups Are Valued by the State

The fourth mechanism for the generation of social value is the capacity of health systems to communicate values by indicating the extent to which various population groups are valued by the state. Because healthcare and other public services are the site of a large proportion of citizen’s daily experiences of the state, and because the outputs of health policy make visible the states’ prioritisation of scare resources across inequitable societies,^31^ the system signals the “value the state…places on different people.”^83^ For example, Reinhardt suggests that by paying providers in a sector intended to serve the poor less that what is considered appropriate payment in a sector predominantly serving the rich, the purchaser, in this case, the state, signals that the health of the poor is less valuable than the health of the rich.^81^ Similarly, Gilson argues that citizens’ “experience of abuse at the hands of healthcare providers represents a soul-destroying confirmation that they are not valued or cared for by society.”^86^ These claims indicate that as a site in which the consequences of prioritisation decisions are made visible to the public, health systems communicate the values of the state to the public.

### Synthesising the Relational Claims Into a Common Frame: Social Values in Dynamic Networks

After exploring conceptualisations of the relationship between health systems and social values found in HPSR literature, and suggesting that, together, these relational claims suggest four mechanisms by which the health system can generate social value, we now present a synthesis of the relational claims and argue that this points toward an explanatory theory for this capacity of health systems.

**Figure 1:**
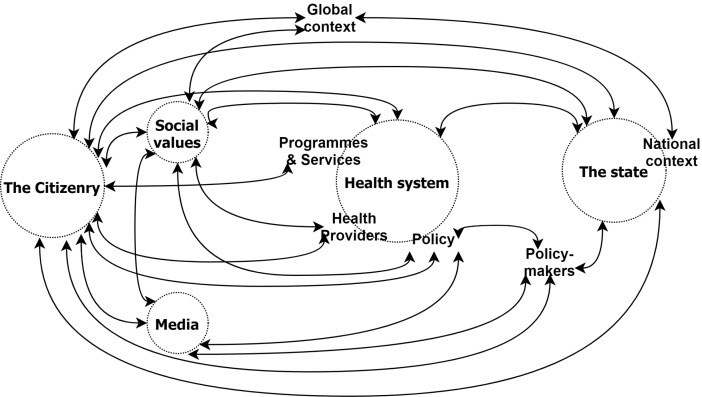
Relational Claims Synthesised Under a Common Frame.

**Figure 2:**
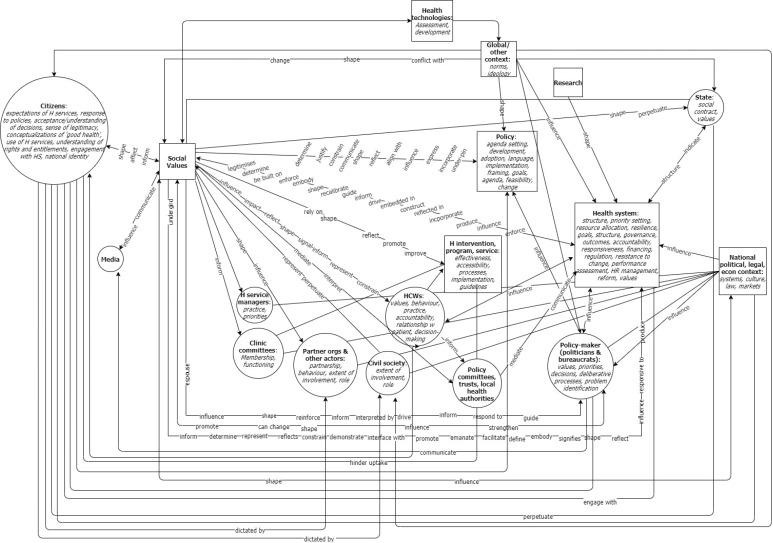
Diagrammatic Synthesis of Relational Claims.

The synthesis, presented in Figure 1, was achieved by combining the relational statements under a single frame in two analytic steps. First, we plotted each relationship claim as a values-based connection between health system components. In order to retain the nuance and complexity of the original conceptualisations, we noted the system functions referred to in each relational claim alongside the relevant component, and noted terms describing the nature of the connection. Each connection between two elements was drawn only once (regardless of how many claims suggested it), and the various functions and characteristics mentioned in the relational claims were grouped under the relevant element of the health system. The direction of influence (where discernible) was indicated by arrows. The resulting diagram is presented in Figure 2.

Because some relational claims posit that social values influence one or more system components, while others suggest a connection between two health systems components that is conditional on, or mediated by social values (as noted above), social values are represented in the synthesis both as a component of the system and in the connections between components.

In addition, because the relational claims are all extracted from HPSR literature, it is not surprising that the idea of the health system as a network of interactions between hardware and software elements of the system (a core concept in HPSR) is common across all the claims. As such, all the types of relational claims discussed already—those asserting a direct causal influence, those suggesting a relationship of constraint rather than enablement, interactions that are conditional on alignment with social values, claims using metaphorical language that suggest dependent relationships, and claims about the influence of health systems on social values (as opposed to the influence of social values on health systems)—can be translated into connections between health system elements.

In the second step, in order to simplify the diagram visually, and aid interpretation of the synthesis, we grouped closely related health system components, removed the functions within each component, consolidated the connecting lines, and removed the descriptors of the nature of the relationship, resulting in Figure 1.

Neither figure is presented as a conceptual framework; the intention is not to simplify complexity for the reader, but rather to synthesise the relational claims within a single frame in order to capture and reflect the full complexity, while allowing the conceptualisations of the relationship between health systems and social values to be considered together.^35^ Nonetheless, the synthesis reveals the dynamic network of interactions between social values and various components of the health system, and the role social values play therein.

Mapping the relational claims into a single frame reveals a complex network of connections not contained within each individual relational claim between health system elements, health systems and the societies in which they are embedded, and health systems and dimensions of the global context. For example, Percival and colleagues’ exploration of social norms that devalue women and girls, suggests that social values operate within health systems to influence the dynamic interaction between HCW behaviour and programmatic outcomes.^89^ Combining this relational claim with others under a common frame reveals that the particular dynamic captured by Percival at al^89^ is also influenced, for example, by health policy-makers’ interpretation of available evidence, itself shaped by social values as demonstrated by Liverani et al^66^ In short, the diagram reveals a dynamic network of values-driven influence between health system components.

Synthesising the multiple relational claims under a common interpretive frame also demonstrates the possibility for dynamic interaction between health systems and features of the national social and political context, such as laws, economic trends and the media. For example, George et al argue that, in Brazil, the country’s history of authoritarianism undermined the functioning of community health councils,^90^ an idea echoed by McCoy and colleagues’ claim that “the political, social and cultural features of society” shape popular attitudes towards community participation in health.^91^ This connection—between social and political characteristics and the functioning of public participation fora—exists in dynamic interaction with, for example, the strength and legitimacy of formal regulatory and governance bodies, itself acknowledged to be influenced by social values.^46^ The synthesis also demonstrates the role of other social institutions, such as the media and civil society. As Abelson et al note, for example, the media can generate awareness on issues that align with, or conflict with, public values, increasing the likelihood decision-makers are compelled to take those values into account.^92^ In addition the synthesis places both patients and HCWs in their social context, suggesting, for example, the influence of citizens values that may differ from patient values,^93^ and the dynamic interaction between social values, political culture, organisational norms, governance arrangements and management practices in influencing the behaviour of HCWs.^10,94^ In short, synthesising the relational claims under a common frame reveals the intricately embedded nature of health systems in their social contexts.^32,42^

In addition to complex networks of interactions *within* national health systems, and between health systems and their social and political context, the synthesis makes manifest another element of the embedded nature of complex systems: the influence of the global on the local. In some papers, the values-influence of the global is understood as a by-product of the natural uptake of technologies and interventions originating in other contexts. For example, Hanefeld et al suggest that “international humanitarian interventions shape and interact with local values shared by health workers, patients and communities.”^55^ Similarly, Reinhardt argues that as a result of geographic and cultural connections to the United States, as well as shared participation in international trade agreements that enable the export of healthcare products (such as private insurance policies) from the United States to other countries, Canada is at risk of importing a set of values entirely at odds with those embodied by the Canadian health system.^81^

However, the synthesis demonstrates that the flow of medical products and technologies takes place in a context of shifting norms at ideologies at the global level. As discussed, many of the papers that suggest global-national connections in relation to social values, focus their attention on neoliberalist ideologies and their pernicious influence on national health systems. Collins et al describe neoliberalism a “worldwide ideological hegemony” that steers health system reforms toward market-driven approaches,^95^ and Fox and Reich concur that neoliberal reforms were ideologically inspired.^8^ As Lencucha et al note, neoliberal ideologies that “shape the global economic order” may well be contrary to “social and cultural norms that express the right to health,”^96^—suggesting that the relationship between health systems and social values, is itself subject to the influence of shifting values at the global level.

While these examples suggest an influence of exogenous neoliberal values on national health systems, some relational claims go a step further to indicate that the influence of these exogenous ideas on national health systems can lead to changes in national values. For example, in a report on healthcare reform strategies in Europe in the 1990s Saltman and Figueras note that the reform process in many European societies was “influenced by the radical market-oriented thinking of the 1980s” and, as a result, those societies “increasingly perceive healthcare as a commodity that can be bought and sold on the open market”^64^—suggesting that neoliberal ideologies can be internalised into society’s conception of the nature of health and the entitlement to healthcare. Malone explores the role of language, and particularly metaphor, in this transference, and finds that in the United States, metaphors reflecting neoliberal ideologies came to supplant other ways of understanding healthcare, and therefore restrict what policy changes are considered acceptable or appropriate.^97^ Similarly, Walt and Gilson argue that the dominance of neoliberal ideas challenges, and may undermine or destroy, socially accepted ideas of “public purpose, public morality, and public accountability.”^21^ Synthesised into a single frame, these relational claims position national health systems as conduits through which powerful ideas at the global level are transmitted to individuals and communities.

As a synthesis of the relational claims identified in the HPSR literature, Figure 1 presents a dynamic network of interactions between actors, organisations, institutions and processes, spanning local, national and global levels. In other words, it presents the relationship between social values and national health systems as a dynamic network of interactions, embedded within larger (global) systems, and subsuming smaller systems (including local, organisational and interpersonal dynamics) within them.^6,101^ In the next section, we explore how this dynamic network of interactions explains the capacity of health systems to generate social values.

#### Offering an Initial Explanatory Theory: Social Value as an Emergent Product of Complexity

Considering the relationship between health systems and social values in this way reveals a plausible explanatory theory for the social value of health systems. It suggests that the capacity of the health system to generate social value—by offering a unifying ideal, shaping the public’s understanding of their rights and entitlements and the responsibility and legitimacy of the state to meet those obligations, improving popular trust in the state, and communicating the value the state places on various population groups—is an emergent property of a complex system (see Box 2). In other words, the interpretive synthesis indicates that complex adaptive systems theory provides an explanation for how social values operate within health systems, and how health systems in turn generate social values. In this section, we demonstrate how this explanatory theory emerges from this interpretive synthesis.

**Box 2. Systems Thinking and Complex Adaptive Systems Theory**

‘Systems thinking’ considers systems as a network of subcomponents and highlights the connections and interactions between subcomponents and the impact of this interconnectedness on the capacities of the system.

‘Complex adaptive systems theory’ can be understood as a category of systems thinking. As a conceptual tool for understanding the behaviour of complex systems, it posits.

**Emergence:** System characteristics emerge from complex interactions among component parts. The whole is different to the sum of its parts.

**Feedback:** Information loops operate within the system.

**Non-linear causality:** Changes have disproportionate effects. Outcomes of intervention are often unpredictable.

**Openness:** Boundaries are poorly defined. Systems influence and are influenced by larger context in which they are nested.

**Path-dependence:** Systems are constrained by history.

**Self-organisation:** Tend towards equilibrium, an apparent order underlies seemingly random interactions between elements

**Sensitivity to initial conditions:** Features of an initial state of affairs can have powerful effects over time.

**References:** ^2,3,42,98-100^

Complex adaptive systems theory suggests that emergence, along with feedback, non-linear causality, openness, path-dependence, self-organisation and sensitivity to initial conditions, are fundamental characteristics of all complex systems^2,99,100^ (see also Box 2). The emergent properties of a complex system are those properties that arise out of the dynamic interaction of system elements, but which are not possessed by any element within the system.^102,103^ In other words, by virtue of the complexity of interactions between elements of the system, patterns begin to emerge in the system as a whole, allowing the system to have properties that would not result from any one particular interaction between system components.^99,100^ Emergent properties are a function of feedback loops, which occur when interconnections between system elements create loops, giving rise to a circular process of cause and effect.^100,102^

In HPSR, systems-thinking—as an approach that applies complexity theory to health policy and systems (HPS) issues—considers health systems as complex systems, made up of connections, interactions and networks between systems elements, including actors.^2,99^ This perspective accounts for the social nature of health systems, and therefore considers the elements of the system from which complexity arises to include ‘hardware’ elements (structures, organisations, and technologies) and software elements (people, relationships, cultures and values), as well as the influence of the social, political, and economic context on the system.^2,78,104^ Here, we show that interpreting the complex network of interactions that form the relationship between health systems and social values from a systems-thinking perspective accounts for how HPSR authors write about the relationship between health systems and social values.

Firstly, feedback loops and emergence account for the influence of health systems on social values and the ability of health systems to inform popular understandings of justice in relation to healthcare. A number of relational claims proposed a macro-level feedback loop between social values and the health system as a whole. For example, Paton argues that health systems shape ideology, but also, conversely, that ideologies can shape health systems.^105^ Similarly, Sheikh et al state that “values drive people’s decisions within the health system contributing to change, and conversely, system reforms can have impacts on people’s values within the system.”^72^ Van Olmen et al specify two likely feedback pathways, stating that health systems “are shaped by values and…enforce these values, through their structure and the inter-personal relationships.”^106^ Conceptualising the operation of social values within health systems as a complex phenomenon with emergent properties suggests that these value-inputs shape health systems, and that, over time, the health system legitimises these values, which then come to be seen as appropriate, or even necessary. This is explained by the self-organising nature of complex systems—from the dynamic network of individual interactions, “patterns emerge which ultimately inform and change the behaviour of the agents and the system itself.”^3^ So, for example, when Heslop and Peterson say that the structure of the US health system reflects the values only of the corporate elite,^79^ but others such as Schlesinger^57^ and Sage^74^ disagree, it may well be because the influence of the system on society as a whole is such that the values of the system have become, or are becoming, accepted as appropriate or just by the population.

Thinking of values as becoming institutionalised over time through feedback loops also accounts for instances in which social values are seen to constrain system change, as is the case when the current design of health programmes shapes “public views on who deserves to be a beneficiary, to what extent, and for what services” and therefore determines public support for or opposition to new programmes or policies.^51^ For example, in a study exploring provider-imposed access barriers in the context of access to family planning services, Calhoun et al suggest that because providers take community and social values into account in deciding what advice and information to give to patients, they inadvertently reinforce social norms by reflecting community values back to patients.^71^ In such a case, a health systems intervention to counteract pernicious social norms through a public education campaign might have little or no effect if the behaviour of HCWs serves to reaffirm existing norms.

From a more macro perspective, health systems are generally understood to be resistant to change,^107^ and this can now be understood (at least in part) as a result of values being institutionalised and legitimised over time. As Freedman et al state “the status quo implies acceptance of the values that currently drive health and health systems.”^28^ In the same vein, Paton argues that “ideas about what is possible are influenced over time, and that can—over an even longer period of time—lead to those ideas coalescing into an ideology of what is desirable…[causing reformers to] trim not only their legislative ambitions, but also their very way of thinking about the issue.”^108^ On this account, if health systems are complex social systems in which values are enforced, legitimised and institutionalised,^64,76,106,109^ it is because a myriad of interpersonal interactions over time continually reinforce the ideas underlying the status quo, which in turn determines the ‘framework of values’^110^ within which decisions about the future are made. Thus, as a result of its complexity, the system develops path-dependence—the feedback loops become self-sustaining, and the system becomes increasingly resistant to change.

A systems-thinking perspective also helps to explain how health systems can generate social value by presenting society with a unifying ideal. Meynhardt suggests this possibility, using the phrase ‘circular causality’ to describe a process of emergence of social values in which “interactions between different elements (people, groups, etc) leads to the emergence of collective properties (eg, shared worldviews, norms and values) which in turn promote consensus, coherence and orientation in chaotic interactions at a microlevel.”^111^ In other words, the system has the capacity to influence social values with respect to healthcare, and these values are legitimated, institutionalised and, therefore, reinforced over time—thereby generating a consensus that becomes more and more deeply rooted over time. Thus, the Canadian commitment to universalism in healthcare, and the role of the state in providing it^51,68^ (discussed above) might be understood as an emergent property of the country’s health system.

The systems-thinking perspective suggests a similar explanatory mechanism for the ability of the health system to communicate the extent to which various groups of the population are valued by the state. As discussed above, the health system is one of the sites through which citizens regularly interact with the state, providing the state “with one of the most visible outputs of policy.”^22^ The synthesis presented in the previous section captures this relationship insofar as it positions health systems as a mediator of the relationship between citizens and the state—suggesting that information about value judgements flow, through a dynamic network of interactions, between citizens and the state. Over time, therefore, users’ experiences of the health system may well begin to influence the extent to which they feel they are valued by the state, and either strengthen or weaken the state’s legitimacy.

Systems thinking also suggests an explanation for the neoliberal phenomenon mentioned above—that of shifting popular perceptions about the appropriate role of the state in healthcare delivery, financing and governance. As was discussed, in some cases neoliberal values come to influence social values through their institutionalisation in the health system. In other cases, however, the values underpinning national health systems are too deeply rooted to be shifted, and neoliberal reforms are rejected. For example, Harrison and Calltorp write of the Swedish experience that “the electorate and politicians…began to withdraw their support for market-type experiments and neo-conservative ideologies, once it became clear that exposure to market forces could weaken Sweden’s social welfare system…and threaten the country’s historic commitment to social equality.”^45^ The fact that in some contexts neoliberal reforms are adopted, while in others they are roundly rejected, can be explained not only by the unpredictability of complex systems’ responses to new stimuli, but also by the fact that, in complex systems, history matters.^90,104,107,112^ The likelihood of adopting neoliberal reforms depends not only on present conditions, but also on historical conditions.

Within health systems the influence of social values is evident across a myriad of elements, functions and relationships. In addition, health systems play an important social role as *generators* of social value. This paper has proposed an explanatory theory for the capacity of health systems to generate social value. On this account, this capacity is an emergent property of the dynamic network of connections through which values operate within health systems, and between health systems and their social and political contexts. As such, the relationship between health systems and social values is causal, but complexly so. Complex causality, a defining characteristic of health systems and a foundational concept within HPSR,^104,113^ suggests that an effect need not be “linked by a linear and predictable path to a cause,” but rather that an observed effect is likely the result of multiple-interacting causes.^104^

Conceptualising the relationship between health systems and social values as complexly causal, accounts for the ways in which the relationship is commonly conceptualised in HPSR literature. As noted above, where it is presented as causal, the influence of values is usually considered to be one among many influences—ie, one connection within a dynamic network of connections. In other cases, it is presented as conditional on alignment between two sets of values, indicating that the potential influence of values depends on, for example the initial conditions of the system, or the interaction between system components and features of the broader socio-political context. In still other cases, social values are conceptualised as constraining system change—accounted for in this explanatory theory by the fact that values, and their institutionalisation over time, is one of the reasons for the change-resistant and path-dependent nature of health systems.

The idea of complex causality also makes sense of the prevalence of metaphor in the relational claims. As Sturmberg et al suggest, “metaphors are central to the human understanding of complex issues,” because they allow us to subsume conceptually challenging or unfamiliar ideas with familiar, everyday ideas.^114^ As demonstrated above, most of the metaphorical language used in the relational claims took the place of explicitly causal language (such as ‘drives,’ ‘underlies,’ or ‘mirrors,’ rather than ‘impacts,’ influences’ or ‘causes’). It is likely that metaphorical language is so common because it allows authors to imply a complex causal interaction, or a dependence relationship, but not a direct, simple causal connection.

#### Leveraging the Social Value of Health Systems: Practical Implications Accounting for Complexity

This synthesis is necessarily dense, and the explanatory theory, by nature, initial. Current thinking on social values in health systems is nascent, although agreed to be important, and has not been critically interrogated through ongoing dialectical engagement.^7^ We explored the ways in which health systems are understood to be capable of contributing social value to the society in which they are embedded, and argued that this capacity is an emergent property of complexity in health systems. We also noted that complex systems are understood to be path-dependent and change-resistant, and that interventions are likely to have unpredictable consequences. This poses a particular challenge to health system reform efforts, which are often understood to be driven more by values and ideology than by evidence or reason^64,95^ and the policy decision-makers who seek to institute them. Here, we offer lessons for policy-makers and researchers seeking to bring about values-based change in health systems. A summary of lessons for policy-makers and researchers is given in Box 3 and Box 4, respectively.

**Box 3. Summary of Lessons for Policy-Makers**

1. Diffuse values-based change through multiple policies, programmes and interventions across the health system.
2. Take advantage of policy development processes as opportunities for values-based dialogue and consensus-building.
3. Ensure that the language used in policy documents and in public communication reflects values.
4. Act as ‘interpreters’ to ensure that values derived from public consultation and engagement are appropriately reflected in policy.

**Box 4. Lessons for Researchers**

- Develop a disciplinary language that reflects the complex reality of causal connections in health systems.
- Employ synthesis approaches that capture nuance and complexity to inform systems-oriented interventions.
- Consider values as drivers of behaviour and decision-making in actors, but also as important contextual and historical factors.
- Conduct HPSR that has conceptual utility to policy-makers, and that promotes values-based change in health systems.

Abbreviation: HPSR, health policy and systems research.

### Lessons for Policy-Makers

Health systems are change-resistant, in part, because values become institutionalised and legitimised over time. As a result, attempts to influence the status quo by introducing progressive values in one programme or policy, are unlikely to have a substantial effect on the system as a whole. As Freedman et al state, attempting to bring about change by deploying equity oriented policies “around the edges of a system whose structure is profoundly inequitable…will not work.”^28^ This reflects the fact that, that values are communicated to citizens through their interaction with health providers, but also through the structure and organisation of the system as a whole.^76,104,106^ As such, policy-makers should be cognisant that values matter—deeply, and in *every* policy change process. In order to shift the trajectory of the system, values-based change must be diffused *throughout* the system, and should take place through multiple interventions across system components—even in ostensibly technical policy arenas such as financing or technology assessment.^8,77,115,116^ This may require developing a values-based strategy for health system reform used to drive incremental change across health system components.

A second lesson is that the policy-making *processes* matter as much as the policies themselves. Health policy decisions only rarely involve a choice between conflicting social values, but more often require trade-offs between competing values—a process of deciding which value to prioritise.^24,117^ Thus, policy processes should be dialogic sites for deliberation and consensus-building,^118^ involving policy-makers “in partnership with an informed public.”^117^ A number of the papers discuss public participation mechanisms that involve deliberative methods as a way to draw out or make explicit social values,^68,119,120^ but as Bombard et al note, such processes are also an opportunity to *reinforce* social values by allowing for the identification of commonalities across citizen perspectives, or allowing “members to find common ground.”^120^ Rather than simply a process of “securing a negative consensus on the shortcomings and deficiencies to be rectified,” health policy processes should be used as opportunities to build a “positive consensus” about values that “are likely to lead the system to a higher stage of development.”^118^

To do so, policy-makers should pay attention to language. Policy discourse, rhetoric and metaphor has an impact not only on how citizens perceive those policies, but also popular conceptualisations of what is right and just in relation to health policy.^57,97,121^ This entails that pernicious ideologies in policy discourse can become popularly accepted values. In this vein, Schlesinger argues that “policy frames incorporate particular norms of fairness. When goods and services are portrayed as marketable commodities, fairness is defined primarily in terms of individual choice and personal deservingness…[and] these notions of fairness would become the primary way of judging equity.”^57^ However this also entails, that policy-makers and other actors have the power to start to shift dominant values by changing policy discourse.^122^ Freedman et al argue that “the more government signals its values through its decisions, proclamations, speeches, and actions…the quicker such values become normalized and part of the accepted discourse of the society.”^28^ Therefore, policy-makers should pay close attention to language choices in the framing and communication of policies.^123,124^

Lastly, incorporating social values into policy decisions requires policy-makers to act as interpreters of social values. Social values change over time, and this requires that policy-makers be sufficiently in-tune with shifts in national values to understand what policy changes or system reforms are feasible in that particular context, and to formulate resonant rationale for proposing new policies.^29,110^ However, social values are not objective—even when evidence about the public’s values and preferences is available, substantial interpretation is necessary before it can be used to guide policy.^125^ As such, policy-makers should consider themselves *in partnership* with informed publics and incorporate social values, evidence and their own judgements into policy decisions.^117,126,127^ In doing so, however, policy-makers should be wary of the self-regulating nature of health systems and guard against the tendency to allow the status quo to define what is possible or desirable.^108^

### Lessons for Researchers

The lessons for policy-makers require a change in perspective in the form of a values orientation and attention to complexity. HPS researchers can support this shift.

Firstly, HPS researchers working with values must strive to develop a disciplinary language that does not shy away from complexity—in this case explicitly identifying non-linear causal connections and considering the influence of contextual and other factors. While the use of metaphor may be an inescapable part of grappling with complexity, the *choice*ofmetaphor is important, because metaphors are not only a function of how we speak, but also shape how we think and how we act.^114,128^ Using metaphorical language risks obscuring the complex but *causal* nature of the relationship between health systems and social values, and may therefore, inhibit policy-makers and others from considering health systems as levers for positive social change.

Secondly, researchers seeking to synthesise evidence about complex health systems to influence policy processes, should consider synthesis approaches that capture, rather than obscure or simplify, real-world complexity.^113,129^ Health systems are inherently complex and “can only be understood by observing the relations and interactions between the elements, not simply by analysing the system’s elements in isolation.”^2^ In this study, we borrowed methodological tools from meta-ethnography, and synthesised the relational claims by presenting them under a common frame. This allowed us to capture the complexity and nuance present in the original papers, and as a result, demonstrates the possibility for dynamic interaction. This, in turn, pointed toward emergence as an explanatory theory. This approach demonstrates the potential of reviews that seek to capture complexity, and reveal the interlinkages between system components.^113^ Such evidence can then be used to inform “system-oriented interventions.”^113^

Thirdly, this study demonstrates the value of using systems-thinking in health policy analysis to understand the role of values in policy processes. Policy analysts are compelled to pay close attention to the behaviour or health system actors, which is strongly influenced by social values.^19,21^ In addition, “conflicts over values are particularly stark in the health policy arena,”^21^ and therefore health policy analysis presents a wealth of knowledge on the influence of values in policy processes. However, the focus on actors in Health Policy Analysis can mean that consideration of values is restricted to the influence of the values of key actors on policy decisions, and there is a recognised need for more research to understand “the clash of values” that influence health policy processes.^130^

This study demonstrates that a systems-thinking perspective can aid health policy researchers to recognise, and account for, the broader influence of values—including in the influence of past policies, the structure of the health system, and the dominant values and political realities in the context and globally—alongside considering the values of policy actors. For example, while the review did not collect data on the political organisation of countries studied, many of the relational claims suggest that contextual particularities of political organisation will influence the behaviour of system actors and the shape of health systems, and are relevant to understanding the role of values in policy change and system reform.^26,34,42,43,45,66^ This dynamic presents a fruitful potential area for future research using principles of systems-thinking to understand the complex role of values in health policy change in context.

Lastly, while HPSR is, by definition, an applied field that seeks to “strengthen health systems so they can better achieve their health and broader social goals,”^19^ it is important to remember that the value of research to policy-makers is not limited to its capacity to determine the best solution for a particular policy problem.^78,131^ HPSR can contribute to promoting values in health systems by “exploring the societal relevance and purpose of systems,”^78^ and by “shifting the framing of health policy debates, and gradually influencing the nature of dialogue.”^124^ Policy problems and policy processes present considerable complexities in their own right, and research that offers conceptual insights of relevance to policy-problems, and shapes the thinking of policy-makers, can have substantial impact in the long-term.^124,132^

## Conclusion

This paper has presented the results of an interpretive synthesis of HPSR literature on social values to generate a plausible and initial explanatory theory for an observed phenomenon. We have demonstrated that systems-thinking can offer an explanatory theory for the social value of health systems as an emergent property of complexity. In the interpretive paradigm, any interpretation of the evidence is offered as one possible plausible reading of the phenomena being studied.^35^ As such, the account presented here should be judged on its plausibility and coherence as an explanation for the capacity of health systems to offer social value.

Nonetheless, we intend the account presented here to have real-world utility in policy processes and be of conceptual use to policy-makers and researchers.^132^ In offering a way to conceptualise the relationship between health systems and social values, and the capacity of health systems to generate social value, we hope to encourage HPS researchers and health policy-makers to more rigorously consider the potential of health systems to strengthen societies, and the effect their work has in this regard. In addition to aiding policy-makers grappling with values-based change in complex, path-dependent systems, we hope that this theoretical work will be further tested and refined by future researchers. If, by paying attention to values and how they operate in complex social systems, it is possible to use those systems to build stronger, more cohesive and more just societies, then endeavouring to understand how to do so is well worth the effort.

## Acknowledgements

The authors gratefully acknowledge the support of the Alliance for Health Policy and Systems Research, Health Policy Analysis Fellowship. In addition to financial support, the EW received invaluable intellectual guidance from Professor Lucy Gilson, Dr. Zubin Schroff, ASSOC. Prof. Maylene Shung-King and the HPA Fellows on multiple drafts of this paper.

## Ethical issues

This research is the product of a broader study for which ethical approval has been granted by the Human Research Ethics Committee at the University of Cape Town, South Africa.

## Competing interests

Authors declare that they have no competing interests.

## Authors’ contributions

EBW was the primary author and wrote the manuscript. She undertook the analysis under the supervision of JO. JO also provided editorial input and made substantive suggestions regarding the structure of the paper.

## Funding

EBW was financially supported in part by the Alliance for Health Policy and Systems Research, Health Policy Analysis PhD Fellowship Programme, the South African National Research Fund, and the University of Cape Town Health Policy and Systems Division PhD Scholarship.

## Endnotes


[1] Please see Supplementary file 2 for a fuller explanation of elements of the meta-ethnographic approach used in this study.

[2] For a detailed account of the methodology of this review, please see Supplementary file 3.

[3] Sturmberg and colleagues published a number of subsequent papers utilizing this framework, but shifted away from using ‘shared values’ as the central attractor of the vortex, preferring a conceptualisation of ‘core values,’ ie, the system’s “focus or goals” that “remain unchanged in a changing world” and “should be the health of every patient.”^70^


## Supplementary files

Supplementary file 1. Data Extraction Sheet.

Supplementary file 2. Meta-Ethnography.

Supplementary file 3. Data Collection and Sampling for the Prior Mixed Methods Review.
