# Supplementary material for: Towards an Explanation of the Social Value of Health Systems: An Interpretive Synthesis
Source: Int J Health Policy Manag. 2020 Aug 26;10(7):414–29. doi: 10.34172/ijhpm.2020.159 (PMC9056134; doi:10.34172/ijhpm.2020.159)
Supplement: Supplementary file 1 — Data Extraction Sheet. [file ijhpm-10-414-s001.pdf]

Authors	Title	Journal	Relation statements	Verbatim quotes
Roemer, M. I. 1993	National health systems as market interventions	J Public Health Policy	SVs → HS.	"The political ideology of a health system can be scaled along another axis [Entrepreneurial & Permissive, Welfare-Onented, Universal & Comprehensive,Centrally Planned & Socialist]" "l. More precisely, the system is shaped by the wealth of the country and by the political ideology govering its health system" "While marketplace values, of course, are still found in health systems -more in some than in others -the concept of health service as a social right has gained ascendancy in most countries of the world" "Concepts of equity and social justice came to replace a free market philosophy in decisions on many aspects of national health system"
Frenk, Julio. 1993	The public/private mix and human resources for health	Health Policy and Planning	SVs → pub/pri mix. SVs → policy.	"Unfortunately, a lot of the public/private debate has looked only at the pragmatic aspects of such alternatives [public/private mix]. Each of them, however, reflects a specific set of values - an ideology - that must be made explicit." "Indeed, the pathological profile of a population reveals its social organization, its way of distributing wealth, and the real value that it places on equity and social justice."it is necessary, first of all, to make explicit the value assumptions that underlie any policy."individual health constitutes a social value, for it represents an input for economic development and an investment in human capital." "if the benefits of health care are social, then its costs should also be distributed among the members of society. Because of their complexity and political value, the mechanisms designed to achieve this purpose always entail the participation of some collective actor - which in the modern world is almost always the state." "health care enables the population to give concrete meaning to the ideals of equity, social justice"
Mooney, G. Jan, S. Seymour, J. 1994	The NSW health outcomes initiative and economic analysis	Aust J Public Health	SVs should inform priority setting. Dec-makers make decisions on the basis of their values or their perception of comm Vs.	"In reaching decisions about how best to deploy resources, more account should be taken, in a structured way, of community values." "These judgements are normally those of health care decision makers, based either on their own values or as a proxy for the community values."

Frenk, Julio. 1994	Dimensions of health system reform	Health Policy	SVs → HS reform. SVs → Pub/pri mix. HCWs → SVs	"Indeed, different forces have led to health sector reform in various countries [4,5]. Broadly speaking, it is possible to identify four major groups of reasons: economic, political, ideological, and epidemiological." "Closely related to political processes are the ideological reasons for reform, as recent doubts about the effectiveness of state intervention, and a belief in the superiority of markets have led countries all over the globe to reconsider the optimal public/private mix in health care," "Unfortunately, a lot of the public/private debate has looked only at the pragmatic aspects of policy alternatives. Each of them, however, reflects the values that underlie the foregoing principles. " "Health care providers offer the state frameworks to interpret human experience. SInce such frameworks represent alteranatives to magical and religious, the state can use them to legitimize different modernising ideologies, and to exercise control over the population (for exampel in such cases as infectious diseases and mental disorders).
Walt, Gill. Gilson, Lucy. 1994	Reforming the health sector in developing countries: the central role of policy analysis	Health Policy and Planning	SVs → operation of programmes. New values can undermine existing values.	"It had become clear that tbe effectiveness of programmes was influenced by values and culture (both national and international), accountability, morale, and communication, among other things, but that such factors· had been neglected in the belief that better techniques or technologies could by themselves tackle the causes of ill-health" "In the 1980s, however, as neo-liberal ideas began to dominate, health policies moved into a policy arena in which previously accepted values were challenged (e.g. by calls for 'cost sharing' and the promotion of private health care providers). A context in which market values dominate leaves little room for morality, values and f eclings, and may undermine anJ destroy previously accepted, socially constructed concepts of public purpose, public morality and public accountability" "Conflicts over values are particularly stark in the health policy arena"
Ham, Chris. Brommels, Mats. 1994	Health care reform in the Netherlands, Sweden, and the United Kingdom	Health Affairs	SVs shape HS.	"This is in part a response to the perceived shortcomings of planning and management, and in part a reflection of changing political values. Not least, the election of conservative governments and interest in drawing on ideas from business management have been important factors in the introduction of markets into health care."
Frenk, Julio. 1995	Comprehensive policy analysis for health system reform	Health Policy	values consensus facilitate HS reform	"Renewal of the health system involves more than securing a negative consensus on the shortcomings and deficiencies to be rectified [lo]. A positive consensus must be created on specific changes that are likely to lead the system to a higher stage of development. In building a positive consensus, it should be recognized from the outset that every health system reflects a particular set of social values and that therefore reform is essentially a political process. "

Okuonzi, Sam Agatre. Macrae, Joanna. 1995	Whose policy is it anyway? International and national influences on health policy development in Uganda	Health policy and planning	Interventions reflect values. Global values transmited throug interventions.	"the interventions thus largely reflected the values, ideologies, objectives and priorities expressed by organizations in New York, Washington and London, not those of any national political system"
Gilson, Lucy. 1997	Implementing and Evaluating Health Reform Processes: Lessons from the Literature	Report: Partnerships for Health Reform	SVs influence reform content. SVs influence reform process. Pol systems influences SVs. Alignment btwn SVs and pol Vs influence reform outcome/implementation. Incentives influence SVs.	"Health reform is a process of organizational and financial changes based on the social and political values of reformers." "A critical factor influencing the particular components of any reform package are the values guiding reformers and the related objectives of reform, which are generally embedded in the underlying political system. " "Among the critical conclusions about reform that can be drawn from experience of these processes are: that values, actors, and actors' perspectives hold significant importance over the content and process of reform" "Bach's (1994) analysis of the French health system structure notes that it is rooted in the tension between socialist principles associated with universal insurance and equity, and the liberal values associated with patient freedom and clinical freedom;" "Experience indicates that reforms that conflict with the values of society or of health care providers will have a limited and unpredictable impact " "the potential of actors to block reform processes is tied to the balance of power between different sets of actors, which is influenced by the broad economic and historical context as well the way the health system has developed historically, all of which help to explain the particular place of different actors within, and the relative importance of different values to, the health system" "The nature of incentives introduced by reforms is a particularly important aspect of design as they will not only influence the performance of the health system, but also the values shaping it"

Saltman, Richard B. Figueras, Josep. 1997	European health care reform: analysis of current strategies	WHO regional publication	SVs are an infuential contextual factor. SVs influence the principles of the HS. SVs influence possibility/desire for reform. HS organisation is a reflection of SVs. H services reflect SVs. Role of state in HC delivery reflects SVs regarding role of the state. How people understand health rights and entitlements reflects SVs. Accountability mechanisms reflect SVs. SVs ensure HS stability. Organisational forms of HS reflect SVs.	"The most influential of ["the central factors that define the context in which health care reform is being pursued"] include a society's cultural values and the macroeconomic requirements of an increasingly global economy, demographic changes and the role of medical technology, as well as specific health sector concerns." "A health system's present structure and capacity for future change reflect a wide variety of contextual factors. Societal norms and values influence the central principles of the system, as well as the desire for reform." "Across Europe, the technical resources that comprise a health system are configured in a variety of different ways...All these arrangements are strongly influenced by the underlying norms and values of the wider society. Health care services, like other human service systems, mirror the deeply rooted social and cultural expectations of society as a whole." "One key indicator of a society's normative values involves the very nature of health care itself. Some societies view it as a predominantly social or collective good...Other societies, influenced by the radical market-oriented thinking of the 1980s, increasingly perceive health care as a commodity that can be bought and sold in the open market." 'A second set of deeply rooted norms concerns the role of the state in the health sector." "one can speak of five different types of health system accountability: ethical, professlonal, legal, political and financial. While every system has elements of all five, typically one or two will predominate - reflecting the values that are central to a particular national health system." "health systems are part of a broader process by which societies decide on which values to prioritize and institutionalise" "The difficulties faced by democratic sodieties in refroming their health care systems are tied to the complex relationships that exist between society's values, the norms adopted in order to put those values into operation, and the existence of individuals who are autonomous yet dependent on those values...It is a question of maintaining a health care system perceived by the public as a central element of society's core values, while simulateneously introducing necessary changes." "The stability of large social systems such as health care is a result of the coherence that exists between society's values...and the social and physical structure of health care institutions. This structure is composed of the processes, laws and regulations that define how resources and authority are distributed in the health sector, as well as the volume and type of resources available. These resources and the manner in which they are organised are a direct reflection of society's values." "THe interaction between actors in health care delivery is defined by one of four basic models found in most parts of society...Each model emphasizes a different set of values. THe dominant model selected for use with a given health care systems reflects the priorites chosed by society as a whole." "organizational structures produce a vast array of incentives. constraints, nhligations and norms that at any moment reinforce a specific set of values, perceptions and beliefs." "Organisational forms become the legal, administrative and physical structure of the dominant value system."
Steinberg, C. R. Baxter, R. J. 1998	Accountable communities: how norms and values affect health system change	Health Aff (Millwood)	Vs influence how communities react to systems change. Community values shape HS change	"the presence of an identifiable “community” with evident common values had a lot to do with whether strong mechanisms of accountability were evident." "Many communities we observed took collective action to erect barriers to entry for outside firms, out of fear that outsiders will not uphold community values as indigenous entities do." "the lack of accountability mechanisms may be an expression of common conservative values that support a market-based approach to health system change" "Alongside policy and economic forces, community values can be a strong factor in shaping health system change.'

Brown, L. D. 1998	Health reform in America: the mystery of the missing moral momentum	Camb Q Healthc Ethics	Resonance of policies with public values impacts public attitude towards policies.	"In the American context, however, moral argument often works politically against interventionist public policy, redistribution, cross-subsidies, and social solidarity, and in defense of entrenched inequities." "Unless health reform is concretely connected to the values and interests of a critical number of these groups they will continue to spook an ambivalent public."
Mooney, Gavin. Wiseman, Virginia. 1999	A 'constitution'for health services	Journal of Health Services Research and Policy	SVs should drive H serv delivery	"he values should come from the community whose health services these are." "the values ofthe community should drive health services,"
Redden, C. J. 1999	Rationing care in the community: engaging citizens in health care decision making	J Health Polit Policy Law	Deliberative processes can develop values among citizenry. HC systems can contribute to national identity. HS rhetoric can ifnluence citizen expectations re role of the state. Shows that interpretation of rights and entitlements can differ.	"In the United States the dominance of individualistic principles often precludes consideration of collective identities (as rights bearers), which means that debates concerning health system reform bring to the table questions about the flexibility of fundamental American values" "However, the development of a certain civic-mindedness among the citizenry (in the Canadian context this translates as social rights identity and indicates a sense of national pride and shared values), has already been achieved." "Health care is, arguably, Canada's most unifying national symbol: Canadians seem to agree that, as a matter of citizenship, each individual is entitled to a comprehensive range of health services." "The rhetoric of grassroots initiatives, citizen inclusion, and community participation, in addition to the actual changes heralded by community-engagement exercises, have begun to adjust citizens' expectations regarding the role of the state."

Giacomini, M. K. 1999	The which-hunt: assembling health technologies for assessment and rationing	J Health Polit Policy Law	New technologies reflect values and social goals of engineers, constituents and users. Trade off decisions reflect ideologies. Decision making tends to reflect SVs	"Researchers currently take too much for granted when they claim to have revealed “values” by eliciting ranked preferences among ad hoc sets of symbolically complex technologies such as “heart transplants” or “critical care for infants.”" "At issue may not be the workings of the technology itself, but some less tractable question regarding the medicalization of problems or the legitimacy of recipients' needs. “Technologies are expressions of fundamental values” (ten Have 1995: 17); the purposes they serve represent social goals envisioned by their engineers, constituents, and users" "The technology assembly process is not arbitrary, but heavily values driven...The contours of the resulting “technological trade-offs” swerve along divisions created by jurisdictional budget structures such as fee schedules, institutionalized interests such as clinical specialties, ideology about principles such as personal versus collective responsibility for illness and care, and information such as available evaluation research." "Especially at the public or administrative levels of decision making, the focus on effectiveness as an “objective” value obscures— but hardly neutralizes—the values that have both brought the technology to our attention and shaped our expectations and judgments of its effects." "Initially, reformers characterized the list of historically covered services as ad hoc and unrationalized, but their failed priority-setting efforts later recharacterized it as the result of “decades of reasonably commonsense and principled decision making . . . [reflecting] fairly accurately the values and priorities of . . . New Zealanders” (report quoted in Cooper 1995: 805)."
Collins, Charles. Green, Andrew. Hunter, David. 1999	Health sector reform and the interpretation of policy context	Health policy	International norms → HS reform	"HSR in developed, developing and transitional societies has been influenced by the worldwide ideological hegemony of neo-liberal, market-driven and 'New Right' approaches to social, economic, and political change. "
Gilson, Lucy. Doherty, Jane. McIntyre, Di. et al. 1999	The Dynamics of Policy Change: Health Care Financing in South Africa, 1994–1999	Major Applied Research Paper 1, Technical Paper 1	congruence between pol maker Vs and policy Vs influence likelihood of pol success/implementation. SVs→ HS reform.	"the importance of actors or stakeholders and their potential to block reforms - which is itself tied to the balance of power between different actors, often rooted in, and shaped by, conflict over the values and goals underlying reforms;" HS reform is influenced by: "cultural factors i.e. the values and commitments of society as a whole and groups within it; exogenous factors i.e. the events and values outside any one country or system that influence it;" "where the technical design of a financing policy matched the values of policy elites, and the broader political goals with which they were associated, there were fewer barriers to implementation" "policies that matched the values of policy leaders and elites were implemented but those that did not" Factors that have been found to infludence reform include "the underlying contextual factors that shape the values underlying reforms and actors' behaviour, as well as determining the nature of selected reform proposals."

Malone, R. E. 1999	Policy as product. Morality and metaphor in health policy discourse	Hastings Cent Rep	Language of policy → 'vision' or understanding of the good in relation to pol. Degree of resonance btwn pol discourse and political ideology → percpetions of pol options	"metaphors based in the language of economics and business increasingly and invisibly supplant other metaphors in U.S. health policy discourse, and to contribute to the conversation about how this may constrain our vision of the good in relation to policymaking." "metaphorical construction favors certain kinds of policy choices over others by virtue of the kinds of meanings the metaphor carries in this culture and its consonance with current political ideology"
Morone, J. A. 2000	Citizens or shoppers? Solidarity under siege	J Health Polit Policy Law	SVs act against market forces	", market forces operate within a deeply held cultural commitment to collective social welfare. "
Geva-May, I. Maslove, A. 2000	What prompts health care policy change? On political power contests and reform of health care systems (the case of Canada and Israel)	J Health Polit Policy Law	Pol change is value driven, but constrained by power and money.	"these reforms were prompted by power contests and not necessarily by the health care systems' financial symptoms or by sheer interest in social values. We claim that financial symptoms only provide the pretext for the contest, while social values concerns are resolved when the power contests allow for them to be taken up."
Harrison, Abigail. Montgomery, Elizabeth T. Lurie, Mark. Wilkinson, David. 2000	Barriers to implementing South Africa's Termination of Pregnancy Act in rural KwaZulu/Natal	Health Policy and Planning	SVs impact HCW provision and rationing of services.	"Interesting paradoxes also arose with regard to the stronger support for abortion in cases of rape or incest among both community women and health care workers, compared with cases of socioeconomic hardship. This distinction says much about community norms. Rape and incest are considered tragedies beyond the control of women seeking the terminations, thus entitling them to do anything to alleviate the situation. In contrast, socioeconomic hardship is not viewed in this light."
Harrison, M. I. Calltorp, J. 2000	The reorientation of market-oriented reforms in Swedish health-care	Health Policy	Vs are resistant to change, and therefore constrain pol change.	"Conflict between the ideology of market-driven health finance and fundamental social and political values proved an even more powerful force for reorienting the competitive reforms than did interest-group opposition to specific reform programs." ". The electorate and politicians across the political spectrum began to withdraw their support for market-type experiments and neo-conservative ideologies, once it became clear that exposure to market forces could weaken Sweden's social welfare system, reduce public employment, and threaten the country's historic commitment to social equality"

Navarro, V. 2001	Science or ideology? A response to Murray and Frenk	Int J Health Serv	SVs exported via H technologies to other contexts	"Because of the enormous influence of the U.S. government on many international agencies, we are seeing their promotion of dominant U.S. values and practices, including managed care and managed competition. "
Ham, Christopher 2001	Commentary: Values and Health Policy: The Case of Singapore	Journal of Health Politics, Policy and Law	SVs inform HS development. SVs inform policies. SVs are reflected in policies	"Health care systems do not develop in isolation. They are products of the societies in which they are embedded and of the values held to be important in those societies." "there is no risk pooling and savings accounts have been established on the basis that each generation should meet its own needs rather than build up reserves or commitments for future generations. This principle of intragenerational accountability rather than intergenerational solidarity reflects the values on which the Singapore system is based and the emphasis placed on personal responsibility (Ham 1996)."
Waitzkin, H. Iriart, C. 2001	How the United States exports managed care to developing countries	Int J Health Serv	Experts, informants, policy-makers etc espouse a 'common sense' that is ideologically drive	"We found that the rhetoric for the policy changes that are occurring throughout the developing world emphasizes the ideology of the private market as a route to more efficient and accessible services" "World Bank has promulgated an ideology that “health is a private matter and health care a private good”"
Atkinson, Sarah. 2002	Political cultures, health systems and health policy	Social Science and Medicine	Macro-level SVs inform pol implementation at local level. SVs inform how rights and responsibilities are understood. External policies and ideas are reinterpreted in line with SVs. Wider SVs inform HS working at local level.	"Research on decentralised health care in Northeast Brazil demonstrates the critical role played by informal aspects of health system management and the political cultures of the wider context on the implementation of policy and the performance of local health systems." "Social organisation and political culture are characterised by clientelism, paternalism, favouritism and personal-links. Within this kind of political culture, public provision of social services becomes a favour rather than a responsibility and the perception of formal public spaces is as anything but public" "these external ideas, concepts, products and so forth are not merely appropriated but are re-interpreted in the context of local meanings and values. " "The health system is characterised as seamlessly integrated into the workings of the wider political culture of the district; the health reform rhetoric and policy have made little impact."
Axworthy, L. Spiegel, J. 2002	Retaining Canada's health care system as a global public good	Canadian Medical Association Journal	Public HC system = defining attribute of national identity. P of universal access in H system reflects "speaks to" sense of fairness and community	"The principle of universal access based on medical need rather than on ability to pay speaks both to our sense of fairness and to our sense of community". "It has been repeatedly noted that we Canadians regard our public health care system as a defining attribute of our national identity."

Franco, L. M.<br>Bennett, S.<br>Kanfer, R. 2002	Health sector reform and public sector health worker motivation: a conceptual framework	Soc Sci Med	Cultural values among HCWs can impede implementation of reform. Societal culture → organisational functioning	HCWs "could experience a conflict between their own notion of public sector values and messages about working for financial gain" "almost all theories acknowledge three broad classes of internal influences on worker motivation: (1) goals, motives, and values" "In some countries, the very fact of being a public sector worker (or civil servant), particularly at more senior levels, is thought to encourage a set of attitudes and values commonly referred to as a ‘‘public sector ethos’’." "Culture has, at its heart, shared values, which contribute to a type of ‘‘mental programming’’ carried out by family, neighborhood, school, and community." "Organizations with internal cultures and structures not in alignment with the broader societal culture may encounter difficulties in their functioning." "Recognition of the specific cultural characteristics of different country environments may help frame a reform program which meshes better with health worker values," "When health workers feel that the values associated with a reform program are not values to which they personally can subscribe, there is likely to be a disaffection with the reform process and a concomitant lack of motivation."
Tenbensel, T.<br>2002	Interpreting public input into priority-setting:<br>the role of mediating institutions	Health Policy	Values → priority setting. Public values are interpreted by priority setting bodies. These bodies also inform public values. Pol makers/processes can also reject public value inputs as uninformed.	"In virtually every case information gleaned from public input was commissioned by a particular government agency and, most importantly, was designed to be digested by that authority." "The meetings were clearly used as opportunities to evangelise to the public about the serious nature of the issue of inadequate health insurance coverage in Oregon and in the USA more generally" "the OHSC and other bodies like it need not be too concerned with demonstrating that their judgements are objective. The real value of such bodies is that they actually have the capacity to make judgements." "‘digestion’ of complex information, including information about public values, is preferable to a more mechanistic approach to the interpretation of public input." "But information that is raw and that has to be swallowed whole (i.e. undigested by mediating bodies) will probably be regurgitated by the policy process."
Schlesinger, M.<br>2002	On values and democratic policy making: the deceptively fragile consensus around market-oriented medical care	J Health Polit Policy Law	Pol frames incrporate norms of fairness. Frames in pol processes influence public understandings of fairness. Dominant norms in pol discourse can influence publice norms (BUT there is a time-lag - old discourse and frames still have influence).	"Policy frames incorporate particular norms of fairness." "When goods and services are portrayed as marketable commodities, fairness is defined primarily in terms of individual choice and personal deservingness." "One might therefore expect that as market ideas and language become dominant in health policy discourse, these notions of fairness would become the primary way of judging equity" "The historical record also demonstrates the extent to which arguments in favor of pro-market health policy invoke distinctive norms of fairness for allocating of health services" "And the gaps in notions of fairness between elite and public advocates of the market are equally pronounced, threatening the public legitimacy ofeven those market-oriented reforms that can garner majority support among politicians."

Boufford, Jo Ivey. Lee, Philip R. 2002	Health Policy Making: The role of the federal government	BOOK: Ethical Dimensions of Health Policy	H pol goals should align w SVs. Pol systems influence understanding of HS goals. SVs should inform H pol making.	"the role of ethics in policy matters, he continues, is "fundamentally, one of identifying the ends sought in policies, examining the values embedded in these ends, and asking whether they are in keeping with social values."" "incrementalism, pluralism and federalism. These are all powerful characteristics of the American political system that have made it difficult, if not impossible, to "clarify and examine the larger goals of health policies."" "There is broad international agreement that the role of the state is to assure the conditions that allow all its citizens to enjoy the highest attainable level of health. This is clearly a statement of social values that, if accepted, can become a guide for decision making in federal health policy." 'Policy making is a critical function of a national health authority. It involves creating and using an evidence base that is informed by social values, so that public decision makers can shape legislation, regulations, and programs to achieve the agenda of the national leaders."
Kehoe, S. M. Ponting, J. R. 2003	Value importance and value congruence as determinants of trust in health policy actors	Soc Sci Med	Values → trust in HS. Value congruence→trust in H institutions Canadians want HS reform to reflect their values. Emotional relationship between population and HS.	"value importance and value congruence on equal accessibility are found to be important factors explaining variation in all three types of trust" "Examples of egregious violation of trust relationships by individuals and institutions abound around the world and have transformed the landscape in terms of how some individuals relate to institutions" "The propensity for Calgarians to withhold high levels of trust might be due to government having broken an emotional bond between Canadians and their health system."
Giacomini, Mita. Miller, Fiona. Browman, George. 2003	Confronting the "gray zones" of technology assessment: Evaluating genetic testing services for public insurance coverage in Canada	International Journal of Technology Assessment in Health Care	SV should drive coverage decisions	"Citizens may supply guiding values by identifying principles or contributing to their operationalization" "Coverage decisions should be fashioned to fit with enduring values"
Reinhardt, Uwe E. 2003	Resisting US Social Ethics	Health Affairs	heatlh services promote SVs, can change/influence social values relating to HC.	"international trade agreements will serve as the Trojan horse onto whose back U.S. corporations wiil load health care and health insurance services to he sold in Canada, but in whose belly they will smuggle into Canada the social ethics and raw political muscling to which these corporations are accustomed at home"

Roberts, Marc. Hsiao, William. Berman, Peter. Reich, Michael. 2003	Getting health reform right: a guide to improving performance and equity	BOOK	Pol makers can connect issues to SVs in pol agenda setting and pol development. Articulation of SVs behind pol improves accountability. SVs → agenda setting. Vs espoused in H reform debate, can impact SVs.	"Countries differ widely in their levels of economic development, social conditions, political values, disease patterns, and institutional arrangements." "not only do problems vary from country to country, but so do values, goals, and ethics" "Even if you lack material resources, you can still design political strategies that may give you substantial leverage in a policy debate, by wisely using symbols that connect to broad social values." "The DALY index...reflects a particular productivity-oriented perspective that could be inconsistent with social values in a society where, for example, the old are viewed as a source of special wisdom." "It is far more difficult for reformers to be held accountable for the values behind their policy recommendations if they cannot articulate those values clearly." "The general culture of a society also influences the agenda-setting process, by making some topics easy to raise in public and blocking other topics as taboo (Douglas and Wildavsky 1982). These values and beliefs tend to be specific to a particular country's culture and traditions (or to a community within the country), for a particular moment in time." "This case shows how once-taboo topics can become public issues for health reform debate, with wide-ranging political and cultural consequences. Understanding the role of cultural values in the definition and selection of topics for public consideration can help health reformers plan which problems to address and how."
Hanney, Stephen R. Gonzalez-Block, Miguel A. Buxton, Martin J. Kogan, Maurice. 2003	The utilisation of health research in policy-making: concepts, examples and methods of assessment	Health research policy and systems	Pol decisions justified by reference to social values.	"Policy-making can be viewed as involving the 'authoritative allocation of values'" "It has long been recognised that policy-making is a complex process. It can involve scientific knowledge and a range of other factors including interests, values, established positions within institutions, and personal ambitions." "Political decisions are normally justified in terms of social values and understandings shaped in the political arena" "Increasing attention is focusing on the concept of interfaces between researchers and the users of research. This incorporates the idea that there are likely to be different values and interests between the two communities"
Figueras, Josep. 2003	Health system reforms and post-modernismThe end of the big ideas	European Journal of Public Health	SVs → HS	"We should acknowledge that there will never be 'scientific' or 'rational' reforms. The role of politics, ideology and values in what is essentially a social process is not only unavoidable but necessary. "

Gilson, L. 2003	Trust and the development of health care as a social institution	Soc Sci Med	shared values → trust in HS, and btwn HS actors. HS can "build value in society" (see also "committing and enforcing") Funding arrangements also both reflect values and enforce values	"a trust-based health system can make an important contribution to building value in society." "Social and political institutions embodying these norms promote affective trust in societies by committing and enforcing upon all those involved in them a specific set of values" "At a second level, funding and resource allocation mechanisms may also demonstrate norms or values, such as solidarity, fairness and procedural justice" "The trust established between organisations with shared values, such as public and not-for-profit health providers, may, therefore, provide the basis for their co-operation to achieve common goals" "The assumptions underlying attitudes and practices within any welfare system can be seen to reflect the settlement on which it was founded, that is the macro-level relationship established between it and broader societal values through the incorporation of these values into the arrangements and practices ofthe system." "The prevailing settlement underlying a welfare system, however, interacts with, and is shaped by, the changing value base of society."
Vetter, N. 2003	Values in the NHS	J Public Health Med	SVs can inform PS	"Programmes relating to effectiveness of treatment, quality improvement, equal opportunities, patient's rights and rationing health care rely on sets of values."
Floyd, E. J. 2003	Healthcare reform through rationing	J Healthc Manag	SVs → priority setting. SVs → resource allocation. SVs → rationing	"American society places great value on tedrnology, which encourages the development and use of high-tech medical treatments, even when the added benefit of these treatments is small compared to ilieir cost" "The U.S. society's belief in vitalismthat everyone is entitled to a long life of unlimited good health no matter its costs-discourages many Americans from setting limits on care"
Brown, L. D. 2003	Comparing health systems in four countries: lessons for the United States	Am J Public Health	HS principles are underlied by moral and cultural foundations	"The core values of these systems—soli darity, community, equity, dignity—remain intact and surprisingly little disturbed by rising costs and by gloomy forecasts that aging, technology, and the rest are rendering their systems unaffordable. The moral and cultural foundations of universal coverage are missing in the United States,"

Mooney, G. H.<br>Blackwell, S. H.<br>2004	Whose health service is it anyway? Community values in healthcare	Med J Aust	Principles that underlie healthcare services should be based on community values and preferences.	"In eliciting these values, there is an important distinction to be drawn between the community and consumers of healthcare services. The forn1er involves citizens; the latter largely patients." "Equity, for example, may be of little concern to the patient; it is n1ore likely to figure in the mindset of the citizen." "These principles point to more community involvement at the broader level, such as in determining the principles that underlie healthcare services in general, or ,vhat has been called 'a constitution' for health services"
Henry, B. R.<br>Houston, S.<br>Mooney, G. H.<br>2004	Institutional racism in Australian healthcare: a plea for decency	Med J Aust	Performance criteria and funding mechanisms can reflect values.<br>Values in systems and services are driven by values in broader society.<br>Global trends in finance etc can change social values.	"that our health services are "institutionally racist"; and that such racism stems from Australia being, or at least having become, an uncaring society." "Institutional racism "refers to the ways in which racist beliefs or values have been built into the operations of social institutions in such a way as to discriminate against, control and oppress various minority groups". "We believe that any healthcare system is a social institution built on the cultural stance of the population it serves. It follows that cultural values should provide the value base for health services."
Balabanova, D.<br>McKee, M. 2004	Reforming health care financing in Bulgaria: the population perspective	Soc Sci Med	HS financing should reflect dominant social values.	"The reform has been driven by an imperative to embrace new ideas modelled on systems elsewhere, but with little attention to whether these reflect popular values." "The health financing model should reflect the predominant values of society"
Giacomini, Mita.<br>Hurley, Jeremiah.<br>Gold, Irving. et al.<br>2004	The policy analysis of 'values talk': lessons from Canadian health reform	Health Policy	SVs are expressed in policy talk/documents. Values expressed in policy documents present national values. SVs 'undergird' health refrom proposals.	"Most policy analysts would agree that values influence policy goals, decisions, and conduct; values (ideologies, interests, principles, goals, etc.) figure prominently in explanatory models of the health policy making process." "one document ...stated within that, 'Canada's health care system is one of this country's foremost social accomplishments, a core value that helps define our national identity'" "Canadian health reform documents historically show little reflection on the nature or importance of values per se. Authors' reasons for discussing values are neither explained nor apparent in the vast majority of documents. " "Only the National Forum on Health explicitly discusses the nature of values, their definition, and their importance for health reform [4,10]. It defines values as 'relatively stable cultural propositions about what is deemed to be good or bad by a society', [10] (p. 4) and distinguishes values from ethics, beliefs, attitudes, or opinions." "This portrayal of values in dynamic conflict echoes academic views (e.g. from economics, ethics) that consider values as goods that must be weighed against each other in a material world where we 'can't have it all'." "While the contents of Table 1 can be characterized as Canadian values, the categories into which they fall (Table 2) are more universal." "Declared values can be powerful imperatives or toothless platitudes, honestly guiding or strategically misleading."

Gross, Revital. Brammli-Greenberg, Shuli. 2004	Evaluating the effect of regulatory prohibitions against risk selection by health status on supplemental insurance ownership in Israel	Social science & medicine	Regulation can counteract market forces in line with social values. SVs guide policy decision making.	"administrative regulations can influence the structure of supplemental insurance to achieve desired social values" "Furthermore, in Israel, equality and solidarity are perceived as desired values; they guide health policy attempts to ensure an appropriate level of and equal access to health care for all sectors of the population, regardless of social status, religion, ethnic background or financial ability" "Many western European countries that mix public and private funding of their health care systems (e.g., The Netherlands, Germany, Austria, France, Switzerland, Belgium) have deemed regulation of the private health insurance market necessary to ensuring that it remain compatible with prevailing values regarding solidarity in health care." "the Israeli case suggests that administrative regulations can influence the structure of supplemental insurance to achieve desired social values—in this case, equality of accessibility and solidarity in financing between the healthy and the ill.""Public debate in israel has concerned policy options to further promote eqaulity and solidarity in this market"
Franco, L. M. Bennett, S. Kanfer, R. Stubblebine, P. 2004	Determinants and consequences of health worker motivation in hospitals in Jordan and Georgia	Soc Sci Med	Broader society → HCWs through SVs.	"The organization and the individual worker are also part of a broader society that influences their goals and values through community expectations, peer pressure, and social values." "Individual differences related to values are more deeply conditioned by societal values and beliefs. However, changes in organizational culture, management practices, and communication can further shape these. "
Walker, L. Gilson, L. 2004	'We are bitter but we are satisfied': nurses as street-level bureaucrats in South Africa	Soc Sci Med	Instittutions and politics → nx values. Nx values → px bh. Nx values → implementation of policy	"nurses' views and values inform their implementation of health policy;" ". Nurses have also been found to impose their value systems upon their patients." ". To understand these judgements and practices, we need to locate nurses in a wider social and historical context: the professional and personal values of nurses have deep institutional, religious and political roots" "These judgements indicate how nurses' values and worldviews influence their responses to the free care policy, including their assessments of its consequences."
Kapiriri, Lydia. Norheim, Ole Frithjof 2004	Criteria for priority-setting in health care in Uganda: exploration of stakeholders' values	Bulletin of the world Health Organization	SVs inform priority setting processes. HCWs can represent SVs. SVs should inform dec-making	"Developing countries are faced not only with a severe lack of resources but may also have cultural values and characteristics that will influence the criteria they use to set priorities" "However, several studies exploring public values in priority setting found that the public regards health workers as legitimate representatives" "we used the health workers' preferences as a proxy for gaining a better understanding of stakeholders' values." "This illustrates the limitations of using empirical values in decision-making and why it is necessary to test such results for ethical appropriateness and general acceptance"

Kapiriri, Lydia. Arnesen, Trude. Norheim, Ole Frithjof. 2004	Is cost-effectiveness analysis preferred to severity of disease as the main guiding principle in priority setting in resource poor settings? The case of Uganda	Cost Effectiveness and Resource Allocation	HCWs represent SVs in priortiy setting decisions.	"However, several studies exploring public values in priority setting indicate that the public regards health workers to be their legitimate representatives [32,33,2,34,4]. Given the weakness of civil society in Uganda, we regard the health workers' preferences as a first proxy for better understanding the public's values."
Dolan, T. C. 2004	A call to action: Healthcare executives must translate social values into workable healthcare programs	Healthcare Executive	HC execs translate social values into HC programmes	"An imporcanc role for healthcare executives has always been to translate social values into workable healthcare programs."
Giacomini, M. 2005	One of these things is not like the others: the idea of precedence in health technology assessment and coverage decisions	Milbank Q	SVs infl dec-makers. Pol rhetoric has power b/c of shared values. SVs inform pol decisions. Current system determine the "fw of values" within which decisions are made.	"Professed values and goals of the health care system must be kept in focus and related faithfully to this framework [of existing policies]." "The ideal, as well as the feasible, institutional arrangements vary with the jurisdiction. In particular, publicly funded health plans (e.g., Canadian medicare, U.S. Medicare) operate under different incentives and obligations than do private health plans (e.g., in the United States). These differences affect the moral force of precedent-based arguments as well as the framework of values within which precedent-based rationales are formulated."
Mooney, G. 2005	Communitarian claims and community capabilities: furthering priority setting?	Soc Sci Med	Values influencing priority setting can be community values, not only individual values	"Bringing in the community and communitarian values to PBMA priority setting exercises can help to overcome some of the barriers to getting PBMA recommendations implemented." "To the extent that the community is one that is cohesive, seeks to build social capital, values its social institutions and in turn values the community as a community—in essence is communitarian (Avineri & de-Shalit, 1992)— so the prospects for such strengthening of health services principles and priority setting are yet further enhanced."
Gilson, Lucy. 2005	Building trust and value in health systems in low-and middle-income countries	Social Science and Medicine	Px interaction w HS conveys informs relationship w/ society.<br>Alignment btwn individual values and HS LOGIC informs use of services.<br>Financing mechanisms reveal who and what is valued by the system.	"Even when lower income, lower status social groups do access health care services, their (expected) experience of abuse at the hands of health care providers represents a soul-destroying confirmation that they are not valued or cared for by society." "Thiede (accepted for publication) argues that an individual's information and knowledge of the health system's logic, and approval of it, is critical in establishing her access." "Such actions see financing mechanisms as signals of value within the health system—both in terms of who matters and whether profit seeking or caring is prioritised."

Freedman, Lynn P. Waldman, Ronald J. de Pinho, Helen. Wirth, Meg E. 2005	Who's got the power? Transforming health systems for women and children	BOOK: UN Millenium Project 2005	SVs inform pol maker decisions. Values direct HS change. HS imply acceptable norms. HS communicate values.	"policymakers face choices. And the choices they make must be fundamentally grounded in the values and principles that members of the global community have agreed should govern the world that we build together." Standard health sector reform attempts at promoting equity are deployed around the edges of a system whose structure is profoundly inequitable. Until the structure is addressed, the solutions will not work. This does not necessarily mean that a massive, immediate overhaul is necessary. It does mean that values must play an important role in setting the direction of change, even if change is managed and gradual." "The status quo implies acceptance of the values that currently drive health and health systems, even if those values are not often acknowledged or made explicit." "those values are not often acknowledged or made explicit. If the current state of global health is unacceptable, if the status quo needs to be transformed, then consciously identifying and addressing the values that operate in health-related decisionmaking in households, communities, districts, countries, and throughout the world—and the relationship of those values to the distribution of power and resources—will be an essential part of the transformative process. " "values derived from human rights are incorporated into policy and program design and implementation (whether or not the term "human rights" is used" "Equally important, we understand health systems to be a vital part of the social fabric of any society. As such, they "are not only producers of health and healthcare, but they are also purveyors of a wider set of societal norms and values" (Gilson 2003, p. 1461)." "practices in the health system that communicate norms and values, which then shape the experience of both poverty and citizenship." "the feudal values and gender discrimination that characterize the broader society also shape the demeaning treatment received by female healthcare workers—with consequences for their treatment of patient" "the operation of such cultures and values determines what actually happens within the system" "The more government signals its values through its decisions, proclamations, speeches, and actions, and the more transparent and inclusive such decisions are, the quicker such values become normalized and part of the accepted discourse of the society." "What are the values informing the mobilization and allocation of funds and the distribution of who pays for what, where, and when?"
Freedman, Lynn P. 2005	Achieving the MDGs: health systems as core social institutions	Development	HS produce SVs. HS defines people's experience of the state. HS communicate values through provider/px interaction. HS communicate values through structure of HS. HS signals values through treatment of HCWs. HS reform signals values. HS communicates ideas of entitlement and oblication. HS communication of SVs influence public trust in HS.	"Health systems 'are not only producers of health and health care, but they are also purveyors of awider set of societal norms and values' (Gilson, 2003). People's interaction with that system thus defines in critical ways their experience of the state and of their place in the broader society. Health systems communicate and enforce values and norms through many different aspects of their operation." "The ways in which health providers are themselves treated by the system sends equally powerful messages about societal norms and values, and no doubt influences their treatment of patients as well " "Societal values and norms are signalled and enforced not only through inter-personal relationships, but also in the very structure of a health system. " But if we regard health systems as more fundamentally social, culturally embedded, politically contingent institutions, then we need to ask what values and norms does the state signal with this kind of reform? Most importantly for the MDGs, what does it communicate about the poor and poverty, about equity, about entitlement and obligation?" "a system signals the values and norms on which to begin to build the kind of trust on which quality health care so heavily depends"

Bennett, S. Chanfreau, C. 2005	Approaches to rationing antiretroviral treatment: ethical and equity implications	Bull World Health Organ	Clinical guidelines are (sometimes) based on value judgements. Rationing decisions should reflect social values.	"the ethical underpinnings of explicit rationing criteria should reflect societal values." "clinical guidelines for ART can involve value judgements and should be inspected and understood in this light"
Saltman, Richard. Bergman, Sven-Eric. 2005	Renovating the commons: Swedish health care reforms in perspective	Journal of Health Politics, Policy and Law	HS reflects SVs. SVs define current HS. SVs determine feasibility of policy options.	"key dimensions of a country's health care system reflect the core social norms and values held by its citizenry." 'This particular combination of continuity and change has occurred as traditional Swedish values of jamlikhet (equality) and trygghet (security) have been challenged in an environment shaped by an aging population, changing medical technology, and Sweden's integration into the European Single Market." "the Swedish case suggests that core social values tied to national culture play an essential role in defining both the structure of existing health sector institutions and the range of feasible policy options with which to modify these institutions" "The Swedish case suggests the degree to which the basic framework of a country's health care system directly reflects the core norms and values of its population." "These cultural values not only have shaped the initial structure of health system organization but also continue to influence proposed reforms to that structure"
Davies, P. 2005	Economic evaluation and society's health values: price and value are different	BMJ	SVs → resource allocation	"Perhaps the real absurdity is that both health economics and evidence based medicine are ultimately trying to find technical answers (seen as being accurate, numerical, objective, value free) to what are really ethical questions about the values behind our choices."
Frenk, Julio. 2006	Ethical considerations in health systems	BOOK: Constructive conversations about health: Policy and values	HS shape and structure reflects SVs. HS promotes SVs.	"Every health system reflects a series of ethical assumptions. Consciously or unconsciously, explicitly or implicity, these assumptions are expressed in the distribution of healthcare benefits and in the organisation of institutions." "Every attempt to reform the health system must begin by asking which values it should promote." [social inclusion, equality of opportunity, individual and family autonomy, social responsibility] "comprise the most fundamental ethical dimensions regarding the model of society that the health system should reflect and reinforce." "The adoption of specific policies for health system reform must first acheive societal consensus with respect to values, principles, and purposes that have been examined in this essay."

Datye, V. Kielmann, K. Sheikh, K. et al. 2006	Private practitioners' communications with patients around HIV testing in Pune, India	Health Policy & Planning	Value systems → HCW-px interaction, what px can say to HCW	"Practitioners operate within larger societal and moral value systems that clearly influence the content and nature of communication they have with their patients. "
Shiffman, Jeremy. 2007	Generating political priority for maternal mortality reduction in 5 developing countries	American journal of public health	Alignment with SVs facilitates agenda setting	"Research has shown that effective political entrepreneurs possess certain distinct features: they are knowledgeable about the issue, they are persistent, they have excellent coalition- building skills, they articulate vision amid complexity, they have a credibility that facilitates the generation of resources, they generate commitment by appealing to important social values"
Husain, Sara. Kadir, Masood. Fatmi, Zafar. 2007	Resource allocation within the National AIDS Control Program of Pakistan: a qualitative assessment of decision maker's opinions	BMC Health Services Research	Mechanism and organisation of funding and delivery → SV of the system	"allocation of resources is not a value free exercise but is intricately linked with needs assessment, political pressures and even donor wishes" "One participant felt "the social value attached to the program (NACP) can only be given by the public sector""
Makundi, E. Kapiriri, L. Norheim, O. F. 2007	Combining evidence and values in priority setting: testing the balance sheet method in a low-income country	BMC Health Services Research	Priority setting should be influenced by evidence and social values	"Procedures for priority setting need to incorporate both scientific evidence and public values."
Ridde, Valéry. 2008	Equity and health policy in Africa: Using concept mapping in Moore (Burkina Faso)	BMC Health Services Research	SVs → what is understood as a policy issue.	"the failure of the implementation of these policies, in terms of their equity objectives, can be largely explained by the fact that the absence of equity was never seen as a public issue. Yet for any situation to become a public issue many factors must come into play [5], and the question of values is obviously central" "social values occupy a predominant but not exclusive position" in health policy.

Edwards, Nancy. Semenic, Sonia. Premji, Shahirose. et al. 2008	Provincial prenatal record revision: A multiple case study of evidence-based decision-making at the population-policy level	BMC Health Services Research	SVs inform policy making	"However this study also revealed that public health decision makers considered improving health, reducing health inequalities and encouraging partnerships in the provision of care to be more important goals than efficient provision of health services, thus underscoring the role of political values in shaping health policy decisions " "Our study focus falls more closely within the latter definition, which views evidence-based health policy (in contrast to evidence-based clinical care) as the application of best current knowledge to the health needs and values of populations rather than individual patients [3]."
Exworthy, Mark. 2008	Policy to tackle the social determinants of health: using conceptual models to understand the policy process	Health Policy and Planning	Congruence with SVs affects pol feasibility. Policy communities can change SVs.	"for any [policy] strategy to be enacted, it must meet a minimum threshold of: 1. Technical feasibility, 2. Congruence with dominant (socio-political) values." "Moreover, addressing SDH or health inequalities may run counter to dominant values and shifting political values would also threaten further this criterion." "It has become apparent that, over the last decade or so, coalitions of advocates have been forming in many countries around a set of core beliefs (relating to SDH) which are challenging existing dominant values."
Brinkerhoff, Derick W. Bossert, Thomas J. 2008	Health governance: concepts, experience, and programming options	REPORT: USAID policy brief	HS objectives reflect SVs.	"These objectives reflect a particular set of societal values, which many, though not all, countries share."
Bloom, G. Standing, H. Lloyd, R. 2008	Markets, information asymmetry and health care: towards new social contracts	Social Science & Medicine	SVs enable trust in market-based systems, SVs constrain individual interests	"Social norms thus impose a self-enforced order on a market, compelling agents to behave fairly and constrain individual interest while engaging in exchange transactions."
Hyder, A. A. Merritt, M. Ali, J. et al. 2008	Integrating ethics, health policy and health systems in low-and middle-income countries: Case studies from Malaysia and Pakistan	Bulletin of the World Health Organization	Values interact with other factors in a complex process to inform policy making	"Complexity of process in health policy-making and public-health practice: a mix of experiences, politics, evidence, finance, values and ethics all interweave. These are codependent components."

Sabik, Lindsay M. Lie, Reidar K. 2008	Priority setting in health care: Lessons from the experiences of eight countries	International Journal for equity in health	SVs → PS.	"In 1996 the Danish Council of Ethics laid out values that should form the basis of the health service: equality, solidarity, security, and autonomy. " The National health Committee "also used information on public values and opinions, gathered through public meetings" "Principles and procedures are supposed to help ensure prioritization is consistent with society's values and goals for the health care system. In fact, most commissions were established precisely to establish shared values on which prioritization decisions can be based." "One of the stated goals of the New Zealand's Core Services Committee was to ensure that core health services "reflect the diverse needs and values of the population being served"
Teerawattananon, Y. Russell, S. 2008	The greatest happiness of the greatest number? Policy actors' perspectives on the limits of economic evaluation as a tool for informing health care coverage decisions in Thailand	BMC Health Services Research	Pol maker perceptions of SVs influence dec-making.	Policy actors who prioritised severely ill candidates ahead of others, even though their treatment was less cost-effective, also argued that the majority of the public would have the same ethical values and expecta-<br>tions for healthcare rationing." "economic evaluation ignores alternative ethical values that can be held by policy actors."
Teerawattananon, Y. Russell, S. 2008	A difficult balancing act: Policy actors' perspectives on using economic evaluation to inform health-care coverage decisions under the Universal Health Insurance Coverage scheme in Thailand	Value in Health	SVs → (reinforce) pol-maker attitudes. Pol makers take alignment with SVs into acc in dec-making.	Pol Maker "attitudes toward economic evaluation must be understood within wider contexts, in particular their organizational allegiances and the institutionalized “ways of doing things” within these organizations or among their social and professional groups, which are reinforced by the norms, rules, and values of those groups or wider society." "In their opinion, the public's acceptance of economic evaluation would be limited if the societal values of equity or justice were not incorporated into decision-making."
Ruger, J. P. 2008	Ethics in American health 1: ethical approaches to health policy	American Journal of Public Health	Hpol reflects values	"more focus has been placed on factors such as procedural mechanisms than on understanding how individuals and groups value different aspects of health and agree on health related decisions." "Health policies reflect the ethical values that underlie them, yet few policy analyses have explicitly assessed these values."
Giacomini, M. Kenny, N. DeJean, D. 2009	Ethics frameworks in Canadian health policies: foundation, scaffolding, or window dressing?	Health Policy	Values discourse/language is commonly included in health policy documents	"While the concepts of ethics, values and principles have become familiar elements of policydevelopment, there is sparse health policy ethics scholarship into their perationalization and application"

Abelson, Julia. Miller, Fiona A. Giacomini, Mita. 2009	What does it mean to trust a health system?	Health Policy	HSs contribute to the construction of broader social values.<br>Interaction btwn citizens and HS inform relationship btwn citizens and state.<br>Dominant HS SVs change over time.	"Because publicly funded health systems comprise such a large degree of state-citizen interaction, mistrust of health systems may contribute to a general mistrust of government. In this way, the health system's contribution to the construction of broader social values and trust, specifically, flows directly from the interaction between citizens and their health system." "the era of paternalistic professional–patient relations has been replaced by the current mode of entrepreneurial customer centered relations."
Lavis, John N. Wilson, Michael G. Oxman, Andrew D et al. 2009	SUPPORT Tools for evidence-informed health Policymaking (STP) 5: Using research evidence to frame options to address a problem	Health Research Policy and Systems	SVs → Pol feasibility.	"A policy or programme can be deemed to be an appropriate solution if it is technically feasible, fits within dominant values and the current provincial/national mood, and is acceptable in terms of budget workability and the likely degree of political support or opposition "
Buse, Kent. Dickinson, Clare. Gilson, Lucy. et al. 2009	How can the analysis of power and process in policy-making improve health outcomes?	The Official Journal of the International Hospital Federation	Congruence between SVs and policy values → likelihood of getting on agenda.<br>Nx Vs influence who they view as deserving px	"nurses' values, including their perceptions of deserving or undeserving patients, had significant implications for the manner in which the free health care policy was implemented in practice." "The technical feasibility, public acceptability and congruence with existing values were all judged to be favourable, and so the three streams came together, putting essential drugs on the policy agenda and resulting in far reaching and radical policy."
Sage, W. M. 2009	Solidarity: unfashionable, but still American	Hastings Cent Rep	Popular values shape HS. But also HS reform can 'recalibrate' public values	"However, explicit solidarity has long been out of vogue in America's value system, despite persistent lack of affordable medical care. Instead, the public has prized scientific innovation, consumer sovereignty, and personal autonomy, and has installed physicians as benevolent oligarchs to oversee these functions. The resulting system delivers idiosyncratic care at enormous expense to most Americans, while a sizable minority often goes without." "political positions and recalibrate public values in support of solidarity."

van Olmen, Josefien. Criel, Bart. Van Damme, Wim. et al. 2010	Analysing health systems to make them stronger	BOOK. ITG press.	SVs should determine national priority setting. HS goals should be based on social values. HS are based on SVs, and the structures of the HS are set up to enforce those values. SVs inform HCW bh. SVs drive the identification and analysis of problems.	"The variation in interpretation and valorisation of values and principles and the underlying tensions result in major challenge to decide on common goals and values in a HS. The values at stake and the balance are unique in each context. Priority setting should take place at country level, in view of technical and rational criteria and broader societal values, whereby existing power balances cannot be ignored." "Strengthening the overall system capacity requires the coordination of efforts based on a coherent policy, vision and long-term view, clearly linked with goals and values;" "a HS should be geared towards outcomes and goals and that HS are and should be based on values and principles" "The interpretation of goals and the choice for a balance between goals is a reflection of the interests, and the underlying values among actors inside a country or local health system. " "The three goals already imply that HSs are not only mechanical structures to deliver health care, but that they are social institutions, implying that they are shaped by values and that they enforce these values, through their structure and the inter-personal relationships." "The choices and priority setting should take place at country level, and take into account technical and rational criteria as well as the values that impregnate the HS and society." "Motivation and commitment are important determinants of health worker performance. Both are influenced by intrinsic personal drivers and external factors, such as management practices, (organisational) culture and societal values." "The analysis of the problems and the identification of priorities are partly technocratic processes, but also strongly coloured by the underlying values of the actors involved."
Sturmberg, J. P. Martin, C. M. O'Halloran, D. 2010	Music in the Park. An integrating metaphor for the emerging primary (health) care system	J Eval Clin Pract	Shape of HS should be responsive to SVs	"Shaping and maintaining a local health care system needs to take account of ever changing patient needs and values."
Kruk, Margaret E. Freedman, Lynn P. Anglin, Grace A et al. 2010	Rebuilding health systems to improve health and promote statebuilding in post-conflict countries: a theoretical framework and research agenda	Social science & medicine	Resource allocation indicates govt values. HS design, and HS financing indicates govt values. HS design, and HS financing legitimises certain values. HS can produce social value.	"How the government deals with inequities in health and how it allocates overall health care resources provide the population with insight into its values and priorities" "One line of this work focuses on how the design of a health system, and particularly its financing, conveys important social and political values of the state. For example, the levying of user fees that are beyond the means of the poorest, legitimizes the exclusion of some portion of the population on the basis of ability to pay (Gilson, 2003)." ". In addition to being an effective platform for delivery of life-saving services, health systems communicate values, intentionally or not. Designing the strategy for the rehabilitation of the health system with specific attention to the health system's political, social and capacity-building functions may help national governments and international development partners to harness the potential gains in social cohesion and rebuilding of trust that are critical to statebuilding."

Rosen, Laura. Rosenberg, Elliot. McKee, Martin. et al. 2010	A framework for developing an evidence-based, comprehensive tobacco control program	Health research policy and systems	SVs → H Pol content	"The first step in the planning process is to agree on the overarching goals. While this may seem obvious at the outset, it is actually complex as it must take into account societal values and political preferences, and should be consistent with broader health policies. "
Pearson, Melissa. Zwi, Anthony B. Buckley, Nicholas A. 2010	Prospective policy analysis: how an epistemic community informed policymaking on intentional self poisoning in Sri Lanka	Health research policy and systems	SVs → Pol framing	"The solutions they proposed offer the opportunity to change things from within agreed values, discourse and interests [36]. If more radical change is needed or desired then this community would need to engage in a wider process to generate support for such options." "The SUPPORT tools also help to provide some guidance on additional areas that are necessary when framing options: technical feasibility fits with dominant values, acceptable in terms of budget and acceptability to stakeholders"
Stafinski, Tania. Menon, Devidas. Marshall, Deborah. et al. 2011	Societal values in the allocation of healthcare resources	The Patient: Patient-Centered Outcomes Research	SVs are embedded in resource allocation decisions.<br>Dec-makers take SVs into account in resource distribution decitions.<br>SVs should inform resource allocation decisions.	"healthcare organizations have made considerable efforts to improve their acceptability by increasing transparency in decision-making processes. However, the social value judgments (distributive preferences of the public) embedded in them have yet to be defined" "an analysis of non-technical factors or social value statements considered by technology funding decision making processes in Canada and abroad" "Social values may be defined as the public's preferences for the distribution of healthcare among populations" "There is little debate among decision makers over the need to ensure that value judgments used to inform decisions (such as consideration of equity or severity) reflect those of the public"
Bombard, Y. Abelson, J. Simeonov, D. et al. 2011	Eliciting ethical and social values in health technology assessment: A participatory approach	Soc Sci Med	SVs → HTA	"growing consensus that ethical and social values should be addressed in health technology assessment (HTA) processes,"having physician incentives built on shared values is the ideal approach to shaping physician behavior" "t is only recently that ethical and social values have been considered by HTA agencies" "Public engagement offers an informed and participatory approach to eliciting ethical and social values in HTA." 'Deliberation about the use and diffusion of new health technology fostered a process of making public values explicit"
Sheikh, Kabir. Gilson, Lucy. Agyepong, Irene Akua. et al. 2011	Building the field of health policy and systems research: framing the questions	PLoS medicine	Ideas, interests, values, norms and relationships interact with hardware elements to inform policy decisions. HS research shapes HS values.	However, in addition to these concrete and tangible expressions of health systems, the ''software''—by which we mean the ideas and interests, values and norms, and affinities and power that guide actions and underpin the relationships among system actors and elements—are also critical to overall health systems performance." "health systems and policies are artifices of human creation, embedded in social and political reality and shaped by particular, culturally determined ways of framing problems and solutions [7,8]. " "HPSR has previously played an important role in exploring the societal relevance and purpose of systems and interventions, and helping shape systems values"

Berlan, David. Shiffman, Jeremy. 2011	Holding health providers in developing countries accountable to consumers: a synthesis of relevant scholarship	Health policy and planning	SVs influence HCW bh, and make HCW bh resistant to change	"alter their behaviour. On the other hand, such beliefs may be so deeply ingrained—a function of both norms in society and professional training—that simple interventions involving information provision may have little impact."
Harris, Bronwyn. Nxumalo, Nonhlanhla. Ataguba, John E. et al. 2011	Social solidarity and civil servants' willingness for financial cross-subsidization in South Africa: Implications for health financing reform	Journal of public health policy	Pol systems shape SVs that constrain HS reform. Shape of the HS reflects social values. SVs inform citizen perspectives on H pol.	"Social solidarity is not neutral as it is embedded within social norms and values. The apartheid system, for example, produced a divisive, but powerful, 'us and them' solidarity centered on 'the myth of the Afrikaner nation and the need for civilized order in the face of the "communist onslaught"'." "The anticipated NHI offers one opportunity for moving the 'thin', or top-down, solidarity enshrined in policies, legislation, and the constitution to a 'thicker' bottom-up form of social solidarity located in the values, relationships, and citizen experiences." "Black Africans indicated greater willingness to cross-subsidize others than whites. This suggests racially or perhaps 'culturally' different values or belief-systems about social relationships, "
Gilson, Lucy. Daire, Judith. Patharath, A. et al. 2011	Leadership and governance within the South African health system	South African health review	SVs inform HCW and decision-maker goals. HS signifies to public what is valued by the system.	"Outcomes pursued by public sector leaders are those judged as valuable by the public at large as well as by political stakeholders and policy makers" "Personal values guide the daily decisions leaders make and the behaviour they role model to others." "Macro-level financing and structural changes, such as those envisaged in relation to NHI, set parameters for health system functioning and offer institutional signals as to what is valued within the system."
Ssengooba, F. Atuyambe, L. Kiwanuka, S. N. et al. 2011	Research translation to inform national health policies: Learning from multiple perspectives in Uganda	BMC International Health and Human Rights	Lack of alignment with values can hinder uptake of interventions. Values espoused by high level decision-makers can also undermine uptake of interventions.	"In addition policymakers contested the SMC research evidence mostly due to concerns such as political feasibility, cultural values and discomfort with complex messages." "Our results show resistance to uptake of SMC due to the nature of the intervention, as well as the underlying values espoused by high level decision makers in the government."
Sturmberg, J. P. O'Halloran, D. M. Martin, C. M. 2012	Understanding health system reform - a complex adaptive systems perspective	J Eval Clin Pract	Shared values influence bh of HCWs throughout the system	"We introduce the concept of the health care vortex as a metaphor by which to understand the complex adaptive nature of health systems, and the degree to which their behaviour is predetermined by their 'shared values' or attractors."

Docherty, M. Cao, Q. Wang, H. 2012	Social values and health priority setting in China	J Health Organ Manag	Values "underpin" health policy	"The primary value underpinning current health policy is clear: all individuals, regardless of their means should be able to access basic health care."
van Olmen, Josefien. Marchal, Bruno. Van Damme, Wim. et al. 2012	Health systems frameworks in their political context: framing divergent agendas	BMC Public Health	HS 'emanate' SVs. SVs as the link btwn context and HS decisions. HS shaped by SVs.	"it views health systems explicitly as social systems that are embedded in a context that shapes its design and development and that in turn emanate the prevailing values of the society to which they belong" "it emphasizes that choices made in steering of health systems are de facto based upon a context-specific balance in values and principles, and thus by power relations between stakeholders" "health systems as social institutions that are shaped by societal values and at the same time act as social determinants in themselves"
Littlejohns, P. Weale, A. Chalkidou, K. et al. 2012	Social values and health policy: a new international research programme	J Health Organ Manag	PS decision reflect civic culture. PS decisions will be different based on different SVs in each country. SVs → PS	"healthcare systems in various national settings will make different decisions about priorities, differences based on the distinctive balance of social values in each country...These differences will also reflect the distinctive civic culture that prevails." "social values in practice shape and influence priority setting." "questions of social value arise in very different institutional and cultural settings."
Gilson, Lucy. 2012	Health policy and systems research: A methodology reader	Alliance for Health Policy and System Research: WHO	HS policies reflect social values.	"financing mechanisms not only influence the level of funding available for the health system, but also indicate what is valued by that system." "financing mechanisms not only influence the level of funding available for the health system, but also indicate what is valued by that system. Here is an example: the taxation-based elements of the system signal the extent to which society is prepared to take collective action to support redistribution; whilst the level of fee for service within the system signals the extent to which society values choice, allowing those who can afford to, to pay for health care to buy more or better services. The set of financing mechanisms, more-over, influence relationships between the state and its citizens as well as between providers and patients, and has a direct influence over levels and patterns of health care utilization, the extent to which the health system offers financial protection in times of health crisis and the contribution of the heath system to generating social solidarity (Gilson et al., 2008)."

Gilson, Lucy. 2012	Health systems and institutions	IN BOOK Health Systems in Low- and Middle-Income Countries: An Economic and Policy Perspective	State should structure HS in line with SVs. SVs inform bh of HCWs and users. HS demonstrate SVs. SVs inform understanding of rights and entitlements.	"government's regulatory role is noted to include structuring the system in line with social consensus on the ethical principles (e.g. ability to pay or social rights) on which it is founded" "the more recent governance frameworks give clearer attention to the norms and values that underpin systems" "All three bodies of theory presented here affirm the relational nature of health systems and the wide range of institutional influences embedded within them. The drivers of actor behaviour go beyond rules and financial incentives to include their relationships with others, the wider set of norms, values, and, importantly, shared meanings that underpin those relationships, and conflicting interests and relative power." "Health system governance must, thus, seek to strengthen the critical processes through which norms and values are demonstrated," "The dominant institutions underpinning [HS] relationships are...the rules, norms and values that confer responsibilities and rights. These can be...informal, reflected in behavioural patterns (e.g. trust, reciprocity, civic-mindedness)" "the population...participates in decision-making with health care providers, or about them. The nature of these exchanges suggest that the underpinning institutions are likely to comprise economic incentives, the rules of decision-making and the norms and values demonstrated by each actor through the experience of decision-making. Health care providers, for example, not only deliver care to the population, but also offer frameworks for interpreting human experience to patients." "the state's relationship to the population involves, on the one hand, offering the subsidies, information, and ideologies that shape population interactions with the health sector and, on the other hand, is based on the basic eligibility principles on which the health sector is founded (which vary between countries from purchasing power, to poverty, to the socially perceived priorities accorded particular population groups, to citizenship). The relationship between the state and the population is thus itself influenced by the prevailing sociocultural norms or consensus that is embedded in these principles." "government's regulatory role is noted to include structuring the system in line with social consensus on the ethical principles (e.g. ability to pay or social rights) on which it is founded"
Clark, S. Weale, A. 2012	Social values in health priority setting: a conceptual framework	J Health Organ Manag	Decisions about recourse allocation should embody SVs	"Social value judgments might be defined as judgments made on the basis of the moral or ethical values of a particular society. Social values can be contrasted with purely moral values in so far as the latter may include values which are not held by a particular society at a particular time" "decisions about resource allocation should embody values which are, broadly speaking, the same values as those held by the population served by the healthcare system in question."
Atun, Rifat. 2012	Health systems, systems thinking and innovation	Health Policy and Planning	SVs → HS functioning, SVs → HS outcomes, SVs → programme success	"A number of factors influence ways in which health systems achieve good health efficiently. These factors include the capacity of both individuals and institutions within health systems, continuity of stewardship, ability to seize opportunities, and contextual characteristics such as path-dependency, sociocultural beliefs, economic set up, and history of the country concerned " "Lessons emerging from instances of low innovation adoption suggest that when addressing health problems, reductionist and linear approaches that provide technical solutions alone are not adequate to mount effective responses, as the adoption and diffusion of innovations which underpin responses to health problems are influenced by complex health systems, the socio-political context within which the health systems are embedded and the innovation adoption system."

Witter, Sophie. 2012	Health financing in fragile and post-conflict states: what do we know and what are the gaps?	Social Science & Medicine	HS financing reflects SVs	"the design of a health system, and particularly its financing, conveys important social and political values of the state." "However, health financing arrangements can convey important messages about political priorities and values."
Kieslich, K. 2012	Social values and health priority setting in Germany	J Health Organ Manag	SVs guide decision making re priority setting.	"values rooted in the political and national traditions of a country affect the organisational form that institutions take...vice versa social values and ideas may be subject to gradual change as institutions develop" "Social values underlie health care decisions in Germany" "social values such as the principle of solidarity and a trust in the self-governing structures form the guiding principles of the health care system and currently present a barrier to health priority setting as it is understood in other national settings."
Ahn, J. Kim, G. Suh, H. S. Lee, S. M. 2012	Social values and healthcare priority setting in Korea	J Health Organ Manag	Nature of sector (pub vs pri) → values (autonomy over solidarity)	"In terms of other content values not explicitly used in Korean healthcare decision making, historically autonomy has been valued higher than solidarity in Korean system probably related to the strong presence of private sector in healthcare system."
McCoy, David C. Hall, Jennifer A. Ridge, Melanie. 2012	A systematic review of the literature for evidence on health facility committees in low- and middle-income countries	Health Policy and Planning	SVs impact how citizens engage with H services.<br>SVs impact how citizens engage with H systems.<br>Citizens can shape SVs through engagement with HSs.	"The political, social and cultural features of society can influence, for example, local community dynamics and attitudes towards CPH" "In rural Nepal, Sepheri and Pettigrew (1996) described how Nepali social norms and kinship relationships influenced community participation, while cultural beliefs and practices about illness and healing influenced the nature of engagement between community members and health service providers. Finally, it is necessary to note that while communities and clinics are shaped and influenced by broader social, cultural and political forces, CPH is also potentially a mechanism for citizens and local actors to shape societal values and attitudes."

Tantivess, Sripen. Pérez Velasco, Román. Yothasamut, Jomkwan. et al. 2012	Efficiency or equity: value judgments in coverage decisions in Thailand	Journal of health organization and management	SVs may compete with eachother in dec-making processes. Value judgements drive decisions about benefit packages. But SVs interact with other factors.	"Social values determined changes in the UC plan in two steps: the development of coverage decision guidelines and the introduction of such guidelines in benefit package formulation." "the prioritisation of benefit candidates was not led solely by evidence, but also by value judgments, even though transparency was recognised as an ultimate goal of reform." "While a number of existing studies have discussed the implications of incorporating social values into health prioritisation, and others determine the relative importance of certain values in hypothetical scenarios in particular societies (Golan et al., 2011; Youngkong et al., 2010), none of these articles provide any comprehensive insight into the role of social values in real-life health resource allocation" "Despite potential conflicts with each other, the social values in the two categories may drive the reform of the UC benefit package development on two levels. First, they dictate the process guidelines that govern the benefit package formulation, given the NHSO's desire to ensure good governance. However, to understand whether and how these ideals were translated into practice, an examination similar to the one carried out in this analysis is needed. The second level of policymaking for which social value judgments can have influences is related to the appraisals of individual technologies." "the roles of these content values in policy decisions are debatable. Policymakers and stakeholders could apply these ideals to support the adoption of cost-ineffective technologies and/or those with substantial budget requirements" "policy decisions are debatable. Policymakers and stakeholders could apply these ideals to support the adoption of cost-ineffective technologies and/or those with substantial budget requirements"
Littlejohns, P. Yeung, K. Clark, S. Weale, A. 2012	A proposal for a new social values research program and policy network	J Health Organ Manag	SVs instruments can and should support health policy decisions	"Country-specific set of values interpretations could function as guiding principles for HTA decision makers in designing socially responsive processes and socially responsive assessments of individual intervention"
Littlejohns, P. Sharma, T. Jeong, K. 2012	Social values and health priority setting in England: "values" based decision making	J Health Organ Manag	Resource allocation dec-making processes reflect national legal systems and values. Dec-making processes reflect and are influenced by SV.` SVs can be embedded in or disguised as considerations of effectiveness.	"While countries are seeking to achieve similar outcomes from their health prioritisation processes, each country has established different systems that reflect the social and legal framework underpinning their health systems." "Social values are also relevant to the policy processes by which priority-setting decisions are reached, since procedural values affect the perceived sense of legitimacy of decisions." "The document "Social values judgements" forms the basis of NICE advice to its advisory bodies on how to apply social-value judgements when making decisions. Both the process and outcomes of appraisals are thus reflective of and receptive to contemporary values and ethical principles held by society." "Social values not only inform the content of the decisions made, but have also influenced the way the process has been set up. " "There are social values being discussed here, but in the guise of judgements on effectiveness"

Mou, Haizhen. 2013	The political economy of the public–private mix in heath expenditure: An empirical review of thirteen OECD countries	Health policy	SVs inform H financing (pub/pri mix). H financing reflects SVs.	"The more ideologically left-leaning the electorate is, the larger the share of public health expenditure" "the choice between public and private funding sources for health care is influenced by the ideological position of the electorate and the voter turnout rate. The public–private mix of health expenditure depends not solely on a balance of interests of voters but also reflects the values deeply rooted in the citizens as a collectivity." "the more left leaning the political ideology of the voters is, the larger the share of public health expenditure in total expenditure. Collective values and social believes are indeed important in the politics around the public–private mix of health expenditure."
Abelson, J. Bombard, Y. Gauvin, F. P. Simeonov, D. et al. 2013	Assessing the impacts of citizen deliberations on the health technology process	Int J Technol Assess Health Care	HTA should be informed by SVs (bc is it is a complex and contentious issue. Not purely techinical)	"Although the CRPHT was established with a clear purpose—to provide MAS and OHTAC with social values and ethics perspectives to inform their analyses and deliberations— OHTAC members presented conflicting views about the kinds of issues they thought the Panel should weigh in on and the boundaries for their deliberations." "At a macro level,the Panel's input had the effect of raising awareness about a range of societal and ethical values relevant to all five technologies reviewed." "The most important contribution of the CRPHT was to "increase the awareness at MAS and OHTAC about the importance of public values, and the importance of seeking the public's opinion."
Kringos, D. S. Boerma, W. G. van der Zee, J. et al. 2013	Political, cultural and economic foundations of primary care in Europe	Soc Sci Med	Value systems affect policy makers' healthcare system priorities; medical professionals' behaviour towards patients; patients' healthcare use and expectations	"Several studies have shown that differences in society's values may explain variation in healthcare policy priorities, services delivery, healthcare utilisation and outcomes" "As the creation of strong PC is a long-term process, there is likely to be a time-lag between changes in political, economic, values or healthcare system contexts to have an effect on PC strength."
Paton, C. 2013	Disciplining health policy? Explaining health policy by reference to...what?	Health Econ Policy Law	Values explain cross-national policy difference. SVs shape HS. HS shape SVs. HS shape bh.	"popular attitudes or 'values' may be taken as autonomous 'inputs' into the explanation" [of the difference between USA HS and UK NHS]. "comparing the United States with mainland Europe and indeed Canada, one perceives a difference in attitude on the part of the majority towards collectivism and individualism in access to, provision of and financing of health care." "Institutions shape ideology. And conversely, ideology can shape institutions" "Institutions shape behaviour" "individuals come to accept prevailing institutional norms as inevitable reality [but also sometimes] reject these norms"
Liverani, Marco. Hawkins, Benjamin. Parkhurst, Justin O. 2013	Political and institutional influences on the use of evidence in public health policy. A systematic review	PloS one	SVs inform decision making processes. SVs influence the use of evidence for health policy. Alignment of of evidence with SVs influences uptake of evidence in health policy.	"Relevant political and institutional aspects affecting the use of health evidence included...the framing of evidence in relation to social norms and values." "It is also well established in social and political science that decision-making processes involve tradeoffs between competing interests and values" "A set of studies were identified that analysed how existing normative positions including: values and moral convictions [31,48,62–64], religious and cultural identity [65,66], or nationalism [46,67,68] can bias the selection or interpretation ofevidence used for health policy development." "political and institutional factors ... have... been identified in the existing literature as important in shaping the uptake of evidence for health policy making. These factors include ...the alignment of evidence with predominant values or existing political agendas."

Leonard, David K. Bloom, Gerald. Hanson, Kara. et al. 2013	Institutional solutions to the asymmetric information problem in health and development services for the poor	World Development	SVs constrain possibilities for change. SVs shape individual and org bh.	"The evidence suggests that macro contextual factors such as cultural norms and values matter for service outcomes, particularly on how they determine the performance of community accountability mechanisms, on how they shape provider–recipient relationships and in the repertoire of well-performing organizations (including government) available" "Thus the organizations that have invested in the creation and maintenance of quality are more likely to have had pre-existing “other-regarding”/altruistic values." "These institutions encompass formal and informal societal and organizational arrangements, incentives, rules, norms, and values that shape the behavior of market actors." "For us “institutions” encompass both—at the market level, there are “macro”/contextual “rules of the game”, whereas at a more “micro” level there are formal policy instruments applied to govern the operation of specific parts of the market, and less formal values that produce and are reproduced by the ways in which particular organizations behave"
Fattore, Giovanni. Tediosi, Fabrizio. 2013	The importance of values in shaping how health systems governance and management can support universal health coverage	Value in Health	HS values impact HS governance. Governance should align with HS values. SVs shape HS.	"The values that are implied by universal health coverage underlie choices about ‘‘how’’ health systems are governed and their organizations are managed. " "governance and management solutions are not independent they are expected to serve. Instead, they should be designed to be consonant with these values. Cultural theory suggests—and experience supports—the idea that ‘‘group identity’’ is favorable for shaping different forms of social life and public administrations. " "he governance and management of health systems are shaped by values " "he values that are implied by UHC matter in how health systems are governed and how their organizations are managed. Governance and management approaches are not indifferent to the values underpinning health systems goals" "the translation of cultural values and goals into practice requires exceptional efforts to design appropriate decisionmaking arrangements (the essence of governance) and management practices. " "governance and management solutions are not independent of the values of the citizens that they are expected to serve. Instead, they should be conceived and designed consistently with these values."
Cleary, Susan. Molyneux, Sassy.Gilson, Lucy. 2013	Resources, attitudes and culture: an understanding of the factors that influence the functioning of accountability mechanisms in primary health care settings	BMC health services research	SVs might not align with system Vs. SVs govern interpersonal interaction within HS. System values are reflected in resource flows. Accountability mechanisms interface with SVs.	"While system level values and organisational culture are related to individual beliefs and values, individual actors would not always share the values and beliefs advocated by the system." "Several empirical papers [24,25,35,36] and review articles [6,20] highlighted the potential mis-match between the values and beliefs of the health system and local communities relative to the democratic and participatory values that underpin external accountability mechanisms" "while accountability processes might be operationalized within interpersonal relationships, these relationships in turn are governed by a series of beliefs that operate at the social or organisational level which structure ways of being and ways of interacting. " "The three wheels presented in the figure suggest the inter-relationships between these elements – where the values of the system are reflected in resource flows; resources and capacity impact on attitudes and perceptions; and attitudes and perceptions impact on the use of capacity and contribute to enforcing or changing values." "How do the values , beliefs, and culture of system actors interface with the accountability mechanism? How does the system and accountability mechanism interface with the values and beliefs of citizens and patients?"

Calhoun, Lisa M. Speizer, Ilene S. Rimal, Rajiv. et al. 2013	Provider imposed restrictions to clients' access to family planning in urban Uttar Pradesh, India: a mixed methods study	BMC health services research	social norms inform provider decision-making. SVs influence how providers interact with px.	"Most providers felt that the social and community norms that put pressure on newly-wed couples to prove their fertility by having a child immediately after marriage were too strong to suggest an alternative."
Fox, Ashley M. Reich, Michael R. 2013	Political economy of reform	Book: Scaling Up Affordable Health Insurance	SVs shape HS reform. SVs shape agenda setting	"Analysis of the political economy of health fi nancing reform shows that there is no consensus about what constitutes a "good" reform, because of disagreement about underlying social values " "A country's dominant political ideas and ideology also shape which policy designs are given serious consideration. (Political scientists refer to this set of beliefs and values in a society as "political culture.") "
Hughes, David. Allen, Pauline. Doheny, Shane. et al. 2013	Co-operation and conflict under hard and soft contracting regimes: case studies from England and Wales	BMC health services research	Social norms influence effectiveness and working of contracts	"We contend that policy designs that emphasize hard market levers, such as fully-specified ('complete') contracts, formal dispute resolution procedures, financial penalties and pay-for-performance incentives, underestimate the importance of informal social norms and long-term relationships in real markets, and risk manufacturing an artificial market environment in which actors fall back on informal relationships to keep the system working."
Daw, J. R. Morgan, S. G. Collins, P. A. Abelson, J. 2014	Framing incremental expansions to public health insurance systems: the case of Canadian pharmacare	J Health Polit Policy Law	Media can communicate SVs and influence pol agenda.<br>Media can also influence SVs by promoting Vs of political elite.<br>National identity reflected in HS.<br>Current policies shape SVs and ideas about Rs and entitlements.<br>SVs are shaped by current and previous policies and policy processes.	"News media itself can influence agendas by independently constructing policy narratives or by acting as a communicator for the public's beliefs, concerns, and values " "Media can also act as a conduit for the political elite to communicate and influence the public agenda" "no one in Canada should be denied access to medically necessary drugs. It contravenes our values as a nation" "Canadians strongly support the universal public financing system for hospital and physicians, viewing it as the fundamental cornerstone ofCanadian identity" "Theories of policy feedback do suggest that public support for government programs is partly derived from the design of existing programs that shape public views on who deserves to be a beneficiary, to what extent, and for what services" "the framing game to be played is dependent on the embedded values of the larger health policy arena and how they have been shaped by previous policies." "one may expect frames to center on the need to expand social policies to reflect the values inherent in existing programs (and thus, arguably, society)"
Sheikh, Kabir. Ranson, Michael Kent. Gilson, Lucy. 2014	Explorations on people centredness in health systems	Health Policy and Planning	Policies reflect SVs. SVs as a necessary input to HS change. SVs inform dec-making. HS reforms influence Vs of HCWs and Pol makers. SVs nb for HS evaluation.	"The World Health Report of 2008 has suggested that people centredness is a requisite 'value' of a primary health care (PHC) approach, required to achieve health for all" "Values drive people's decisions within the health system contributing to change, and conversely, system reforms can have impacts on people's values within the system." "Social values also crucially shape identities of people within the health system." "people centred service delivery, meanwhile, as highlighted by Ferrer et al., for instance, once more emphasises the importance of the values and principles of PHC." "Acknowledging the human values linked with people centredness ultimately may also provide a yardstick against which to assess actions and decision in health systems"

Sheikh, K. George, A. Gilson, L. 2014	People-centred science: strengthening the practice of health policy and systems research	Health Res Policy Syst	Values are a context factor that influence HS actor bh	"Broader contextual influences also seep into the daily practice of a health system through the experiences, mindsets, and values that shape the behaviours of the actors within it"
Paton, Calum R. 2014	The politics and analytics of health policy	International journal of health policy and management	SVs inform pol-maker values. Political constraints shape Vs of pol makers	Comparing the US with mainland Europe and indeed Canada, for example, one perceives a difference in attitude on the part of the majority towards collectivism and individualism in access to, provision of and financing of healthcare." "If, for example, there is no chance of a bill to establish an NHS or a comprehensive system of public health insurance passing in Washington, then reformers over time trim not only their legislative ambitions, but also their very way of thinking about the issue." "ideas about what is possible are influenced over time, and that can—over an even longer periodbof time—lead to those ideas coalescing into an 'ideology' of what is desirable (i.e. politics as the art of the possible, where the practitioners eventually internalise the possible as the (most) desirable state-of-affairs)."
Walt, Gill. Gilson, Lucy. 2014	Can frameworks inform knowledge about health policy processes? Reviewing health policy papers on agenda setting and testing them against a specific priority-setting framework	Health policy and planning	SVs → pol framing. SVs → pol feasibility	"Where common issues such as abortion, same sex practices, sex work are closely allied with public discourse on moral values, it may be a rational policy decision to underplay policy dialogue. "
Bloom, Gerald. 2014	History, complexity and health systems research	Social Science & Medicine	SVs make HS resistant to change	"emphasise the importance of beliefs and social attitudes for the stability of a health system and argue that this is one reason why health systems are highly path dependent."
Tashobya, C. K. da Silveira, V. C. Ssengooba, F. et al. 2014	Health systems performance assessment in low-income countries: Learning from international experiences	Globalization and Health	SVs should be taken into account in HS performance assessement. SVs determine HS goals and tradeoffs.	"A number of authors highlight the importance of considering societal values and principles as they vary across societies, yet are crucial in determining system goals and trade-offs" "Societal values and principles determine system goals and trade-offs"

Gilson, L.<br>Schneider, H.<br>Orgill, M. 2014	Practice and power: A review and interpretive synthesis focused on the exercise of discretionary power in policy implementation by front-line providers and managers	Health Policy and Planning	SVs impact HCW bh, and implementation of policy.	"community values and public discourse underpinned South African nurses' refusal to provide abortion" 'social and Islamic norms lead women to be excluded from decentralized decision-making structures in Senegal" "nurses' value judgements about which client groups were and were not 'deserving' provided a source of power (i.e. the justification and confidence) to provide or refuse to provide abortion services." "personal factors can influence policy acceptance and implementation (see Table 3)—and were underpinned by social and professional norms." "sociocultural values and the political and economic context" influences implenetation of policies"
Ruano, Ana Lorena. Sánchez, Silvia. Jerez, Fernando José. et al. 2014<br>Flores, Walter	Making the post-MDG global health goals relevant for highly inequitable societies: findings from a consultation with marginalized populations in Guatemala	International journal for equity in health	SV inform understanding of health. HS and HC can reflect or fail to reflect SVs	"In Guatemala, many community members define health as a holistic concept that includes positive relationships with the environment, neighbors, friends and family [25,26]. However, the country's health system values the biomedical paradigm over community knowledge, and the longstanding power inequalities that affect indigenous and rural populations translate into the exclusion of non-conforming views. The marginalization of these peoples, both at the health system level and in other areas of society, has translated into very poor access to low quality care that does not reflect their worldviews or the values of the culture to which they belong" "Health is seen as 'the good of the people' , and community health is a shared value that comes from the feeling of belonging to a community."
Mc Hugh, Sheena M. Perry, Ivan J. Bradley, Colin et al. 2014	Developing recommendations to improve the quality of diabetes care in Ireland: a policy analysis	Health research policy and systems	SVs influences pol feasibility, and agenda setting	"Table 2 outlines why retinopathy screening was considered a "quick win" proposal, in line with the criteria for survival in the policy stream: technical feasibility, alignment with dominant values, and acceptability in light of future constraints." "Considering the other criteria for survival in the policy stream, the role of a national clinical lead had already been successfully established for cancer care (technical feasibility); hence, the proposal was in line with values in the health system at that time (acceptability)"

Byskov, Jens. Marchal, Bruno. Maluka, Stephen. et al. 2014	The accountability for reasonableness approach to guide priority setting in health systems within limited resources–findings from action research at district level in Kenya, Tanzania, and Zambia	Health research policy and systems	SVs inform priority setting. Priority setting tools aid in the incorpotation of SVs into dec-making.	"However, central in priority-setting decisions are values, which in practice are rarely adequately recognized, made explicit, defined, discussed, and agreed upon. While technical approaches in priority setting may be useful and necessary, they have been questioned. First, they are based on health and economic data and do not take social, cultural, and other values into consideration." "Decision-making approaches that do not permit discussion and choice on the basis of relevant values tend to produce disagreement, a low sense of ownership, and controversy around both the desired outcomes and the allocation of resources." "that are communicated to all relevant parties involved. AFR thus provides decision makers with an approach to consider and jointly discuss competing values in the priority-setting process." Relevance of AFR principles was "was facilitated by the team's shared values – values ultimately based in the Primary Health Care (PHC) strategy which had been implemented some 30 years earlier [34], and which was reflected in their motto "provision of health services in partnership with the community"." "prior to the REACT project, CHMT decisions were largely based on priorities set by the national 'essential health package' with little consideration of local values." "AFR was regarded as a concrete and workable approach to strengthen the influence of values and context on decision making" "However, to transgress actual power relations (authoritative or technical insight-based) would need acceptance that communities and individuals are the experts on their own values, and that technical values only lead to sustainable progress if weighed against the social and individual values that facilitate engagement." "stakeholders are concerned with commonly shared, seemingly universal values pertaining to fairness." "It is the value base that determines the criteria used, the information needed, the priorities set, and, finally, the decisions made"
Russell, Jill. Swinglehurst, Deborah. Greenhalgh, Trisha. 2014	'Cosmetic boob jobs' or evidence-based breast surgery: an interpretive policy analysis of the rationing of 'low value'treatments in the English National Health Service	BMC health services research	SVs inform dec-makers thinking. But values are also socially constructed among those present.	"Meaning was constructed dialogically through local interaction and broader socio-cultural discourses about breasts and 'cosmetic' surgery." "Studying deliberation illuminates how the notion of 'value' is dialogically constructed, and how 'value' judgements are made about who is deserving of NHS funding and who is not." ". Although some researchers have explored 'social values' in health care rationing [31,32], values are usually treated as pre-defined, fixed entities (for example 'process' values of transparency, accountability and participation, and 'content' values such as equity, solidarity and autonomy), that act as an 'objective' checklist to decision-making, rather than as contested, emergent and situated phenomena [33]. Our analysis aligns with those who view the process of ascribing value as an interpretive act involving ethical-moral choices, constructed moment-by-moment by social actors through interaction [34]."
Whitty, J. A. Littlejohns, P. 2015	Social values and health priority setting in Australia: an analysis applied to the context of health technology assessment	Health Policy	SVs like justice/equity might be less explicitly and systematically considered than effectiveness.<br>Process and content of PS decisions incorporates social values.<br>SVs should be incorporated into PS decision making content and processes.	"The term "social values" refers to those values that are considered important by the society that is affected by the decision-making process. " "there is generally little explicit information available on how these social values are derived, how they are considered in decision-making relative to clinical and cost-effectiveness, or what contexts might engender particular values such as equity to be considered." "Priority setting processes for the subsidy of health technologies in Australia incorporate, either explicitly or implicitly, a range of social values related to both the process and the content of decision-making." establishment of [a deliberative engagement] process in Australia would seem a reasonable approach …to encourage legitimate decision-making that is aligned with community values"

Grundy, John. 2015	Health systems and social transition in Asia	THESIS: Deakin University	SVs→HS reform agenda.<br>SVs → HS change.<br>SVs → priority setting.<br>SVs → HS finance.<br>SVs→HR management.<br>SVs →role of private sector and CSOs.<br>**Political superstructure → SVs.**	"Sturmberg et al (2012) indicates that health policy reform agendas are shaped by "attractors", which are defined as the shared values which pre-determine the directions which reform takes." "The cluster of values surrounding the evolution of the political and social systems sets the scene for the construction of different universal health coverage pathways." "Values and political ideologies can be central to policy directions through providing a window of opportunity for change, particularly during political electoral cycles" "Health priority setting in recent years has been dominated by concepts and practices of economic evaluation, but that in fact much of this priority setting is shaped by the values and perceptions of electorates." "Social policy outcomes are still dependent on the dominant values and directives of ruling political elites and on the capacity of disadvantaged groups to successfully challenge the dominant political narratives that exclude or ignore them." "What the case studies have demonstrated is the way that a health system is financed and its human resources are managed, including the scope of participation of middle level management, civil society actors and private sector actors in health, is dictated to by the core values of the overarching political superstructure."
Hämäläinen, Riitta-Maija. Aro, Arja R. van de Goor, Ien. et al. 2015	Exploring the use of research evidence in health-enhancing physical activity policies	Health research policy and systems	SVs → pol-making process	"As most of the policies were prepared by committees or working groups, the members brought knowledge, experiences, practices, networks, and values to the policymaking." "In addition, ideological and political values of members of working groups and committees, such as political parties and their programmes, democratic engagement of citizens, or specific advocacy for certain population groups by the representatives of organisations, influenced policymaking-"
Suphanchaimat, R. Kantamaturapoj, K. Putthasri, W. et al. 2015	Challenges in the provision of healthcare services for migrants: a systematic review through providers' lens	BMC Health Serv Res	SVs → HServices. SVs → community bh.	"Furthermore, this matter is tightly intertwined with several social determinants, which are related not only to migrants' characteristics (such as, different gender roles, cultural diversity, migration experiences, and precarious legal status), but also the contextual environment of migrant destination countries (such as, idiosyncratic health systems and cultural values)" "The model suggested that the success of health care delivery was dependent on the performance and integration of the health system at different levels, namely: (1) individual patient level, (2) care team level (e.g., clinicians, pharmacists, and others), (3) organisation or workplace level (e.g., hospital, clinic, nursing home, etc.), including infrastructure and complementary resources, and (4) societal level (e.g., legal framework, cultural value, and country economics)." "It is undeniable that services for migrants are dynamic and impacted not only by providers' individual attitudes, but also by the health need of migrants and their family members, as well as the influence of underlying health system, legal implication and social values. "
Martiniuk, Alexandra L. Abimbola, Seye. Zwarenstein, Merrick. 2015	Evaluation as evolution: a Darwinian proposal for health policy and systems research	Health research policy and systems	Pol makers promote SVs. Pol makers are influenced by SVs.	"Health policy is political [4]. Policymakers have goals which reflect the social values and interests they are promoting, influenced by public concerns and mass media attention to a problem, and, to a lesser extent, by data"

Jan, S. 2015	Proceduralism and its role in economic evaluation and priority setting in health	Soc Sci Med	Values drive decision making in priority setting and economic evaluation.	"The focus of Mooney's concerns were the values and power relationships that influence decision making in the health sector and often go unquestioned in conventional health economics research, which Mooney tended to label as 'neo-liberal' (Mooney, 2009)." "practising economists routinely invoke values based on the ethics of individualism which by default are built into conventional economic approaches."
Kok, Maryse. Kane, Sumit. Tulloch, Olivia. et al. 2015	How does context influence performance of community health workers in low-and middle-income countries? Evidence from the literature	Health Research Policy and Systems	SVs shape HCW bh. SVs shape how HS respond to interventions.	"Context influences the daily practice of the health system through the experiences, mind-sets, and values that shape the behaviour of actors within it and, therefore, despite similar elements and patterns, they can respond differently to the same new idea, policy, or intervention." "CHWs' initiative can also be positively influenced by social and cultural values. Community volunteer workers in palliative care in Uganda reported that the cultural desirability of and value attached to the act of helping each other underpinned their caring role for sick community members" "We identified social and cultural norms, values, practices, and beliefs as important community contextual factors that affect CHW performance" "Community contextual factors that were found in the literature related to socio-cultural factors (including social and cultural norms, values, practices and beliefs,"
George, Asha. Scott, Kerry. Garimella, Surekha. et al. 2015	Anchoring contextual analysis in health policy and systems research: A narrative review of contextual factors influencing health committees in low and middle income countries	Social Science & Medicine	SVs inform membership and workings of H committees	"There is a wide range of mandated membership mixes in HCs, with some reflecting social norms for elite involvement but others presenting efforts to structure HCs as spaces for challenging the exclusion of marginalized groups. " "In contrast, in Brazil, due to the history of authoritarianism, health council dynamics tended to echo the larger political culture which was about contestation, rather than dialog or collaboration"
Fox, A. M. Reich, M. R. 2015	The Politics of Universal Health Coverage in Low- and Middle-Income Countries: A Framework for Evaluation and Action	J Health Polit Policy Law	Ideology influences financing mechanisms. Neoliberal policies reflect ideological assumptions.	"Ideology particularly affects the progressivity of financing structures and the degree to which the private versus public sector is preferred as the vehicle for service delivery" "Neoliberal economic ideas, for instance, constituted a series of policy recommendations with a strong rightward ideological tilt toward a vision ofa leaner state, with fewer social protections (Williamson 1994)." "Yet neoliberalism was an ideologically inspired model, part ofa wider paradigmshift toward a leaner and meaner state with a greater role for the private sector in public provision (Reich 2002; Williamson 1994; Fukuyama 2004)."
Landwehr, C. Klinnert, D. 2015	Value congruence in health care priority setting: social values, institutions and decisions in three countries	Health Econ Policy Law	Congruence between SVs and Institutional values → public acceptance of prioritization decisions. Design of HS at large 'reflects' social values. Value congruence → satisfaction and acceptance of HS.	"The public acceptance of prioritization decisions, and eventually of the health care system at large, will ultimately depend not only on considerations of procedural fairness, but also on the congruence between a society's values and its institutions." "In the United Kingdom, the support for efficiency and equality criteria is reflected in the institutional design of the health care system at large and in the design of NICE as the central institution in technology appraisal in particular." ' the incongruences between societal values, institutions and decisions found in Germany may be a central cause behind the significantly lower satisfaction with the system:"

Bouwman, Renée. Bomhoff, Manja. De Jong, Judith D. et al. 2015	The public's voice about healthcare quality regulation policies. A population-based survey	BMC health services research	Alignment with SVs improves legitimacy of regulatory efforts	"tripartism can prevent conflicts of values between the different stakeholders. In tripartism, a public interest group participates as a third group in the regulatory process: it is given power by being granted access to all the information that is available to the regulator, and by being offered a seat at the negotiation table for enforcement and compliance" 'it is important for ensuring the legitimacy, public accountability and transparency of a regulator and for involving the public in regulation policies that the values of regulatory policies are consistent with the values of communities."
Saltman, Richard B. 2015	Health sector solidarity: a core European value but with broadly varying content	Israel journal of health policy research	SVs interact with other pressure to determine resource allocation. SVs inform resource allocation. SVs shape service delivery content and process.	"solidarity has long been acknowledged as a core value in European health policy debate." "he particular health service content that solidarity covers - "who gets what, when, and how" to apply Lasswell's famous definition of politics - has varied quite considerably, depending on the political and economic era, on the country's norms and values, and on the particular structure of the country's health-related institutions " "Cultural norms and values also played an important role in shaping the specific services that solidarity should cover. In earlier times, for example, an important component of workers mutual insurance was a death benefit, to ensure that a covered member could afford a proper Christian burial and thus not suffer the ignominy of a pauper's grave." "ain, and 18.4% in Estonia up to 37.4% in Latvia [28]. While all these EU health systems would each still say that they continue to honor the European value of solidarity in the finance of their health care system, the range in out-of-pocket payments among these countries currently differs by as much as seven-fold."
Mladovsky, Philipa. Ndiaye, Pascal. Ndiaye, Alfred. et al. 2015	The impact of stakeholder values and power relations on community-based health insurance coverage: qualitative evidence from three Senegalese case studies	Health Policy and Planning	Users see values embodied in policies and financing arrangements. Alignment of SVs improves user engagement w H services.	"t values and power relations inherent in social networks of CBHI stakeholders can explain levels of CBHI coverage." "There was also a belief among some stakeholders that the values which they saw CBHI to embody (solidarity, trust, voluntarism and poverty alleviation) were not upheld by politicians." "CBHI schemes could decrease drop-out and increase enrolment by bringing CBHI more in line with local values."
Costa-Font, Joan. Forns, Joan Rovira. Sato, Azusa. 2015	Participatory health system priority setting: Evidence from a budget experiment	Social Science & Medicine	SVs inform priority setting processes and decision. SVs inform HS structure. Alignment with SVs improves legitimacy of PS decisions	"Health care decision making under publicly financed systems is a 'community commodity'. In other words, rather than a market allocation, health care decisions in such systems are the result of a 'socio-political market', whereby social exchanges highly depend on social norms (e.g., equal access for equal need) (Heyman and Ariely, 2004) and political processes." "We agree with Franken and Koolman (2013) that the structure of a health system depends in large part on society's choices concerning resource allocation; and hence it is important to elicit ex ante socially inclusive personal preferences in social justice valuations." 'There is growing interest in involving citizens in policy development to ensure that decisions are legitimate, and reflect broad social values"

Pollock, Allyson M. 2015	Morality and values in support of universal healthcare must be enshrined in law: Comment on" Morality and Markets in the NHS"	International journal of health policy and management	SVs reflected in politices. SVs shape social contracts.	"The moral values of the citizens in support of social solidarity were thus transformed into a political and legal contract for citizens." "There was nothing moral about this extraordinary act of savagery and violence against the public in England, and against common values and widely held beliefs in public ownership funding and provision of universal healthcare." "the values of social solidarity that led to the creation of the NHS in 1948" 'The moral values of the citizens were thus transformed into a political and legal contract for citizens."
Nabyonga-Orem, Juliet. Dovlo, Delanyo. Kwamie, Aku. et al. 2016	Policy dialogue to improve health outcomes in low income countries: what are the issues and way forward?	BMC health services research	SVs inform pol deliberations.<br>SVs inform public respnse to policies.	"The process leading to the outcomes is iterative and takes place within the broader health and non-health systems and the broader context of social values and political systems." ". In that situation, hidden power, manifested through the political stance and the resistance of the communities premisedoncultural values, preventedanobjective dialogue."
Cady, P. 2016	A system of system lenses for leadership decision-making	Healthc Manage Forum	SVs → HS. SVs → HS change. HS → value on users.	"the mere existence ofCanada’s publicly funded health system is an indication of deeply held social values, which have given rise to the system itself in a reinforcing manner. But now, Canadian values and beliefs are calling for more systemic change, " "The system adds value to the person entering it."
Weale, A. Kieslich, K. Littlejohns, P. et al. 2016	Introduction: priority setting, equitable access and public involvement in health care	J Health Organ Manag	SVs should → policy decisions	"public participation also raises issues of social and political values that give rise to its underlying rationales. There are a number of strands of democratic theory that suggest that, if citizens take an active interest in matters of public service, then both instrumental and intrinsic values are served." “Public deliberation rests on the democratic principle that important societal decisions–particularly issues involving competing values and complex trade-offs–are best made by policymakers in partnership with an informed public” (American Institutes for Research, 2016).
Seidman, G. Atun, R. 2016	Aligning values and outcomes in priority-setting for health	J Glob Health	Vs are prioritised by pol-makers, and therefore have important implications for health spending, impacts on populations. Values influence decision–making	"the values and outcomes prioritized by policy-makers have important implications for health spending and the impacts they have on populations and countries." "health policy in countries such as Turkey, the UK and Brazil has influenced the political landscape and political outcomes" "Values and beliefs already influence decision–making; as discussed later, policymakers should make these values public and transparent to other decision–makers and the public in order to legitimize their decisions and to invite productive debate."

Davy, Carol. Harfield, Stephen. McArthur, Alexa. et al. 2016	Access to primary health care services for Indigenous peoples: A framework synthesis	International journal for equity in health	HS that are responsive to SVs improve accessibility. Alignment of SVs between services and px is a social determinant of health	"Broadening models of care to encompass a more holistic sense of health including aspects of social, emotional and cultural wellbeing was considered important." "The types of cultural determinants of health that influenced Indigenous peoples' ability to access health care related to the ability of the health care service to understand and take account of local beliefs and values when providing care."
Chanturidze, T. Obermann, K. 2016	Governance in Health - The Need for Exchange and Evidence Comment on "Governance, Government, and the Search for New Provider Models"	Int J Health Policy Manag	HS Governance must 'incorporate' and 'be built on' 'shared cultural values and norms'	"...the issue of shared cultural values and norms. Governance concepts cannot be transferred, they need to be built on what is culturally appropriate." Good goverance models must incporporate "the prevailing cultural values and norms of a society."
Whyle, Eleanor Beth. Olivier, Jill. 2016	Models of public–private engagement for health services delivery and financing in Southern Africa: a systematic review	Health Policy and Planning	HS represent/reflect national values. Financing mechanisms can reflect or align with national values. Values can shape/strengthen partnerships	"Furthermore, the influence of donors is not always in keeping with national public health priorities, national social values or health system strengthening." "the disbursement of donor funds can be subject to value-based conditions, such as PEPFAR grants, for example, which at times have been dependent on explicit opposition of prostitution (Carter et al. 2006). As such, reliance on donor funding can inhibit the ability of health systems to be representative of national priorities or values (Biesma et al. 2009), undermining national ownership." "The mechanisms by which partners exert control can be formal—utilizing formal contracts and financial incentives, or informal/relational—using interpersonal relationships, industry norms, reciprocity, values and trust to manage the behaviour of partners."
Mostafavi, H. Rashidian, A. Arab, M. 2016	Health Priority Setting in Iran: Evaluating Against the Social Values Framework	Glob J Health Sci	SVs should inform pol makers decisions	"health policymakers...should act according to ethical and subjective values of the public" Most Pol-maekrs "stated that they had never precisely thought about social values in the health sector."
Rispel, Laetitia C. de Jager, Pieter. Fonn, Sharon. 2016	Exploring corruption in the South African health sector	Health Policy and Planning	SVs shape bh of HS actors	"Rational choice forms the basis of agency theory and as such a normative criticism of agency theory is that it is 'outward-looking', it does not account explicitly for broader social values and how trust, for example, could decrease the problems identified by agency theory" "'Inward-looking' solutions, such as steps to instil values of trust and social cohesion might bemissed."

Polisena, Julie. Burgess, Michael. Mitton, Craig. et al. 2017	Engaging the Canadian public on reimbursement decision-making for drugs for rare diseases: a national online survey	BMC health services research	SVs can guide decision-maker deliberations	"Public engagement helps to inform decision-makers about the diverse perspectives of the Canadian population, including differences among stakeholder, patient, and citizen perspectives" "Since it is necessary to consider the fair distribution of health services or funds across health needs and how that is to be weighed against access to DRDs, it is important to include citizens or public whose interests are outside the access to DRDs." "Wider representation may add legitimacy to decisions and policies by involving diverse citizenry on what counts as fair and reasonable and by adding transparency to decision-making processes" 'societal values can be introduced to guide discussions and increase transparency on reimbursement decisions."
Abelson, J. Allin, S. Grignon, M. et al. 2017	Uncomfortable trade-offs: Canadian policy makers' perspectives on setting objectives for their health systems	Health Policy	Public Vs inform decision-makers (particularly around trade-offs)	"The public's values toward different health issues was also viewed as an important consideration in determining priorities, particularly when the media shine a light on specific problems." "trade-offs were described as 'tangly political questions' that are at their core 'value choices'. They were also viewed as constantly evolving and shaped by the policies of the current government or a story that appears in the media, which attracts attention to a particular issue, or other societal values."
Yang, Qian. Pan, Jay. 2017	Control under times of uncertainty: the relationship between hospital competition and physician-patient disputes	International journal for equity in health	Structure of HS influences rel between HCW and px. SV (personal control) interacts w/ HS structure to influence rel between HCW and px (disputes).	"The ultimate success of China will depend on its ability to create stable, efficient rules and regulations which create feelings of control and certainty for people to cope with social pressures, and to upgrade the equality and quality of health care services. Many countries all over the world are in the midst of adjusting the structure of their health care markets. The role of government control and free market competition are two important ideologies....No matter which method is chosen, the possibility of medical disputes can be decreased by guaranteeing people's feelings of control.
Watt, Nicola. Sigfrid, Louise. Legido-Quigley, Helena. et al. 2017	Health systems facilitators and barriers to the integration of HIV and chronic disease services: a systematic review	Health Policy and Planning	SV shape interaction between HCWs and Px. [coded as bidirectional rel btwn citizens and hcws, and influence of SVs on citizens and hcww]	"genuine implementation can be constrained by competing management priorities and social norms shaping the interaction between providers and population" "Although the focus of the review was on health system-related factors affecting integration, the results also highlight the importance of patient preferences, family support, social norms and culture (Isaakidis et al. 2013) in integration of services. This is a reminder of how the patient has agency in shaping the treatment interaction, reflecting the concept of people-centred systems"
Rodriguez, D. C. Whiteside, A. Bennett, S. 2017	Political commitment for vulnerable populations during donor transition	Bull World Health Organ	CSO → SVs. SVs → pol commitment and resource allocation.	"Local civil society organizations can play a role in changing norms and accountability in various ways." 'Sustainable political commitment for key populations may require the changing of social norms and the improvement of accountability, two long-term processes that donors can support but not impose." " We need more evidence on what has and has not worked in mobilizing and protecting key populations around the time of donor transition. We need retrospective research on: (i) the changing of social norms, "

Renmans, Dimitri. Holvoet, Nathalie. Criel, Bart. et al. 2017	Performance-based financing: the same is different	Health Policy and Planning	Framing either resonates or conflicts with SVs. SVs influence design of H financing/pol	"Thus, the wider definition may raise awareness among policymakers that the choice for PBF is only the beginning of the decision-making process and does not inevitably lead to a ‘neo-liberal’ turnaround of the health care sector. This is important because ideological, cultural, social and political values matter and, thus, politics matter. This also indicates a third research pathway: to elaborate how ideological inclinations and cultural values influence the design of a specific PBF scheme, but also whether and, if so, how PBF can contribute to different kinds of policy objectives (e.g. a more ‘neo-liberal’ or a more communitarian organization of the health sector)."
Mirzoev, Tolib. Kane, Sumit. 2017	What is health systems responsiveness? Review of existing knowledge and proposed conceptual framework	BMJ Global Health	SVs shape citizen expectations of HS. SVs are NB for responsive HS	"responsiveness has been described as a principle of wider governance and an outcome of relationship between the people and the state or service providers." Public expectations are "shaped by characteristics of what services are available, their perceived quality and trust and the sociopolitical societal views on health as a human right."
Abimbola, Seye. Negin, Joel. Martiniuk, Alexandra L. et al. 2017	Institutional analysis of health system governance	Health Policy and Planning	SVs → HS goals. HS reflects values. HS values facilitate or hinder accountability. SVs → resource flows. Resource flows shape SVs. SVs cause bh to deviate from prescribed. SVs → px-HCW relationship.	"Fattore and Tediosi (2013), based on the work of Hood (1998), described how the dominant cultural and societal values may influence the decision to aspire to achieving universal healthcare coverage: fatalist values, hierarchist values, individualist values, and egalitarian values." "three factors influencing accountability; the first is a hardware component—resources (including time, space, and especially capacity), and the other two are related to health system software—attitudes and perceptions of health system actors; and the values and culture of the health system. The relationship between these components is that “the values of the system are reflected in resource flows; resources and capacity impact on attitudes and perceptions; and attitudes and perceptions impact on the use of capacity and contribute to enforcing or changing values” (Cleary et al. 2013: 8)." "This body of work has highlighted for example, the need to understand that the way in which practical norms deviate from formal rules reflects both traditional values, and the weakness of governments in the provision and oversight of public goods and services." "In addition, their review also highlighted that in these settings, ‘macro-contextual factors such as cultural norms and values matter for service outcomes, particularly on how they determine the performance of community accountability mechanisms, [and] on how they shape provider–recipient relationships (Leonard et al.2013; 82)’."
Gilson, Lucy. Lehmann, Uta. Schneider, Helen. 2017	Practicing governance towards equity in health systems: LMIC perspectives and experience	International Journal for Equity in Health	SLB bh signals value state places on people. SVs influence governance and practices of SLBs.	"Some papers, meanwhile, specifically illuminate how ‘intangible software’ such as values, norms, communication practices and power or trust relationships, influence everyday governance practices and are themselves situated in wider political and organizational contexts and histories." "SLBs are, quite simply, the daily reality of the state in most people’s experience and so their behaviours signal the value the state, society places on different people’"

Fusheini, Adam. Marnoch, Gordon. Gray, Ann Marie. 2017	Stakeholders perspectives on the success drivers in Ghana's National Health Insurance Scheme–identifying policy translation issues	International Journal of Health Policy and Management	Vs underlying pol should 'resonate'/ reflect SVs.	"Home-grown health financing systems that resonate with social values will also need to be found in the process of translation." "Principles underpinning the NHIS ensure it resonates with the citizenry as it is in line with certain established societal values, norms and customs. Many participants likened the health insurance to the "Nnoboa"[4] the Akans[5] concept associated with and other groups. It is a communal process, where members of a community come together in a show of solidarity to support each other for the benefit of the entire community. The concept of insurance translates to this concept through the pooling of funds and spreading of risks so is not entirely new or alien to Ghanaians." "The Ghana experience illustrates that in adopting health financing systems that function well, countries need to customise systems to suit their socio-economic, political and administrative settings. Home-grown health financing systems that resonate with social values will need to be found in the process of translation."
Hampshire, Kate. Porter, Gina. Mariwah, Simon. et al. 2017	Who bears the cost of 'informal mhealth'? Health-workers' mobile phone practices and associated political-moral economies of care in Ghana and Malawi	Health Policy and Planning	SVs → pol decisions. Global norms → Policy.	"Governments and international donors have often been quick to adopt morally imbued arguments to justify minimal payment (or 'compensation') of CHWs " "the Ethiopian Government justifies their non-payment by positioning CHWs as 'so valuable that they became "priceless" and therefore only remunerable with immaterial satisfaction' " "This moral framing of health-workers in general, and CHWs and volunteers in particular, is also closely associated with a wider neoliberal development discourse that has long promoted 'community participation' and voluntarism as routes to self-reliance. "
Rutstein, S. E. Price, J. T. Rosenberg, N. E. et al. 2017	Hidden costs: The ethics of cost-effectiveness analyses for health interventions in resource-limited settings	Glob Public Health	Values determine benefit of intervention in context	"Use of a common utility weight that does not consider differential life expectancy and health expectations may inappropriately categorise the utility decrement of an illness based on resource- rich setting values, distorting the potential benefit of interventions when applied to resource-limited settings"
Mladovsky, Philipa. Ba, Maymouna. 2017	Removing user fees for health services: a multi-epistemological perspective on access inequities in Senegal	Social Science & Medicine	SVs inform power relations which exclude px from care, and inform relationship btwn HCW and px	"Arduous bureaucratic procedures also disproportionately affected those who lacked assistance from their children to accompany them to hospital or care for them...the exclusion of old men as part of a wider shift in social values, caused by the empowerment of women and children through modern education." "A lack of economic capital could also be seen as an indirect cause of exclusion from PS due to shifting social values which held wealth to be a source of respect."
Kredo, T. Abrams, A. Young, T. et al. 2017	Primary care clinical practice guidelines in South Africa: qualitative study exploring perspectives of national stakeholders	BMC Health Serv Res	Vs inform clinical practice guidelines.	"CPG development represent a complex network of interactions, informed by values and power."

Tweheyo, Raymond. Daker-White, Gavin. Reed, Catherine. et al. 2017	‘Nobody is after you; it is your initiative to start work’: a qualitative study of health workforce absenteeism in rural Uganda	BMJ Global Health	SVs impact HCW bh (attendance). Governance impacts HCW norms	"A majority of the participants reported that attendance at weddings and burials was socially mandatory, " Social-cultural norms accepting of absenteeism and societal expectations pressured healthcare workers to influence their absence motives" "Unlike the PNFP sector, health system-facilitated absenteeism was more prevalent in the public sector, where more favourable norms accepting of worker absenteeism existed, signifying weak health workforce governance."
Kapiriri, Lydia. 2017	International validation of quality indicators for evaluating priority setting in low income countries: process and key lessons	BMC health services research	Priority setting should reflect SVs	Priority setting should reflect public values as measured by "proportion of decisions reflecting public values, documented strategy to enlist public values, number of studies commissioned to elicit public values"
Cairney, Paul. Oliver, Kathryn. 2017	Evidence-based policymaking is not like evidence-based medicine, so how far should you go to bridge the divide between evidence and policy?	Health research policy and systems	SVs are translated into policy. Pol makers turn their values into policy. SVs can impact services via users	"an often implicit and untested assumption is that policymaking should be as ‘evidence based’ as medicine, which is at odds with the more common starting point, in the study of politics, to produce a democratic system which translates competing societal values and preferences into policy solutions." "[Policy] coalitions will be with many actors with different ways of thinking about policymaking, engaging in politics to turn their beliefs and values into policy, often unaware or unsupportive of EBM’s hierarchy of evidence, and likelier to refer to governance principles other than evidence-based policymaking." "This produces a form of government in which the co-production of policy solutions is based partly on evidence and partly on the principles underpinning local governance, including ‘localism’ (central governments recognise the role of partly autonomous local authorities or partnerships) and service user-driven services (based on their values and preferences) "
Agyepong, Irene Akua. Kwamie, Aku. Frimpong, Edith. et al. 2017	Spanning maternal, newborn and child health (MNCH) and health systems research boundaries: conducive and limiting health systems factors to improving MNCH outcomes in West Africa	Health research policy and systems	Policies [?reflect?] social values. Vs underlie HS. SVs shape HS outcomes.	"Every health system has explicit and implicit underlying values that influence decisions and actions." "We considered the underlying values of health systems to be the pillars of the house or shelter." "we think values drive and shape the outcomes of health systems." "Types of context, or the surrounding circumstances within which health systems develop and function, are multi-layered, from global to national through to subnational. They include demographic patterns and trends, sociocultural factors, including societal values and norms, macroeconomic conditions and trends, history, politics and ideology" "However, it could be deduced that equity, as a value, was implicit in the myriad of fee exemptions, community-based health insurance and national health insurance scheme (NHIS) policies that had been and were still being tried in several countries in the sub-region to improve access for the poor." "there was sometimes a clash of values between more economically oriented efficiency perspectives and more socially oriented equity and effectiveness perspectives."

Schaaf, Marta. Topp, Stephanie M. Ngulube, Moses. 2017	From favours to entitlements: community voice and action and health service quality in Zambia	Health Policy and Planning	SVs shape HCW interaction w px. SVs shape HCW bh. SVs enable bottom up accountability	"In addition to sanctions, more normative approaches describe how professional or social norms favoring accountability can engender enforceability." "For example, health care workers provide care, adhere to guidelines, interact with each other and interact with patients according to their personal values, social and professional norms and larger health system infrastructure, among other factors."
Abelson, J. Tripp, L. Sussman, J. 2018	I just want to be able to make a choice': Results from citizen deliberations about mammography screening in Ontario, Canada	Health Policy	Programmes reflect citizen and px values	"Deliberative methods are ideally suited for topics like policy making for mammography screening which are associated with a large and contested body of research evidence, and which involve competing values and trade-offs about potential policy solutions." "Across all deliberations, participants voiced clear support for a greater emphasis on informed decision-making. Values identified that should be reflected in organized screening programs included: choice, information, trust and transparency, financial accountability, and shared decision making with their health care providers"
Petricca, Kadia. Bekele, Asfaw. Berta, Whitney. et al. 2018	Advancing methods for health priority setting practice through the contribution of systems theory: Lessons from a case study in Ethiopia	Social Science & Medicine	alignment of pol with SVs improves implementation. SVs inform process of priority setting.	"Through the application of the TSCF, a nuanced understanding of priority setting practice is revealed that situates this process within a system of interdependent components that include: norms, operations, regulations, and resources" "Through an examination of system norms, Ethiopian social values and cultural beliefs appeared to play a significant role in shaping how priority setting transpired." "Thus, when the principles of a policy have greater congruency with the social and cultural values within a health system, effective implementation is more likely to occur" "the implementation of WBHSP was facilitated by an alignment between the apparent system norms and its overarching principles." "the degree of alignment between system norms and the change effort plays an important role in the implementation of a systems change intervention."
Shankardass, Ketan. Muntaner, Carles. Kokkinen, Lauri. et al. 2018	The implementation of Health in All Policies initiatives: a systems framework for government action	Health research policy and systems	SVs influence policy agenda among pol elites. SVs facilitiate intersectoral collaboration.	"There may also be stronger equity interventions (i.e. interventions that are more upstream), as reflected by the values and ideas implicit in the theory of change for resulting health equity interventions." Part of the executive subsystem is "the cluster of ideas, beliefs, values and attitudes that constitute the normative lens through which political elites and party leaders interpret and act upon social and political issues. " "The cluster of ideas, beliefs, values and attitudes that constitute the normative lens through which policy-makers within a given sector interpret and act upon social and political issues such as health equity, and which may vary given sectoral objectives (e.g. healthcare, population health, economic growth, engineering), i.e. a worldview." can facilititate intersectoral action.

Langlois, Étienne V. Daniels, Karen. Akl, Elie A. et al. 2018	Evidence synthesis for health policy and systems: a methods guide	WHO report	SVs drive pol decisions. SVs influence effectiveness of interventions. SVs are reflected in HS.	"Health policy and system decision-making is also driven by societal values and norms and is largely influenced by stakeholders' interests and power. " "If these types of interventions are to be successfully implemented and scaled up, we need a greater understanding of the factors that may influence their success and sustainability. These include the values, preferences, knowledge and skills of stakeholders, and the feasibility and applicability of the intervention for particular settings and healthcare systems." "Health systems are also social institutions, in that they are both a product of the society in which they occur and an influence on that society (6, 11, 12). The values and principles held by the society are likely to be reflected in the health system; for example, if a society values social equity, it may have a health system that favours universal access to health care"
Sriram, Veena. Topp, Stephanie M. Schaaf, Marta. et al. 2018	10 best resources on power in health policy and systems in low- and middle-income countries	Health Policy and Planning	SVs → pol agenda	"Understanding how and why certain policies and interventions gain more attention than others, and the implicit value judgments underlying those processes and decisions requires an exploration of power dynamics" "The performance of any health system is guided by the interplay between 'hardware' (finance, medical products and technologies, information systems, human resource cadres, nature of service delivery, organizational structures, legislation) and 'software' (ideas, interests, values, norms, affinities and power)". "underlying social and political norms that block or suppress actors from raising issues or taking action." "Bachrach and Baratz (1962) expanded on these explicit dimensions of power by positing that another 'face' of power was non-decision-making;in other words the underlying social and political norms that block or suppress actors from raising issues or taking action. "
Koduah, Augustina. Agyepong, Irene Akua. van Dijk, Han. 2018	Towards an explanatory framework for national level maternal health policy agenda item evolution in Ghana: an embedded case study	Health research policy and systems	In democratic contexts, dec-makers must be seen to be resposive. Shapes policy processes and framing of pol.	"Having to win popular elections every 4 years and retain the mandate to rule have shaped the government health agenda over time. Political actors want to be seen as responsive to social values, institutional and economic challenges, societal, public and international pressures, and as interested to direct new policies and reinforce or remove existing ones."
Lencucha, R. Reddy, S. K. Labonte, R. et al. 2018	Global tobacco control and economic norms: an analysis of normative commitments in Kenya, Malawi and Zambia	Health Policy and Planning	International norms → national pol adoption and implementation	"tension between neoliberal norms that shape the global economic order, and social and cultural norms that express the right to health, universal access to education and other social conditions "

Kruk, Margaret E. Gage, Anna D. Arsenault, Catherine. et al. 2018	High-quality health systems in the Sustainable Development Goals era: time for a revolution	The Lancet Global Health	SVs inform HS. HS shape SVs. SVs shape HCW-user relationship	"High-quality health systems should be informed by four values: they are for people, and they are equitable, resilient, and efficient." "In addition to better health, people derive security and confidence from having a trusted source of care when illness renders them most vulnerable. In this way, health systems also function as key social institutions, both deriving from and shaping social norms and able to promote or corrode public trust." "Patient–provider relationships are shaped by societal norms and are susceptible to power imbalances" "Other research has found that increased technical quality of health services, combined with responsive service delivery, fair treatment, better health outcomes, and financial risk protection, was associated with an increase in the probability of having trust in government."
Bonello, M. Morris, J. Azzopardi Muscat, N. 2018	The role of national culture in shaping health workforce collaboration: Lessons learned from a case study on attitudes to interprofessional education in Malta	Health Policy	nat culture → interpersonal interaction btwn HPs<br>nat culture → uptake of innovations among HPs	"national culture can influence collaboration between different health professional groups"<br>"it is national cultures operating at deeply rooted values level which have an even greater impact on change and innovation"
Percival, V. Dusabe-Richards, E. Wurie, H. et al. 2018.	Are health systems interventions gender blind? examining health system reconstruction in conflict affected states	Global Health	SVs impact HCW bh. SVs impact user BH. HS impact SVs, build stronger societies.	"The lack of respect from health workers, and their abusive and degrading behaviour toward women, emerged as an important factor that undermined their willingness to seek care across all four contexts. This abusive behaviour reflected gender norms across all four settings that devalue women and girls." "Cultural norms emerged as another important factor impeding access to health services." "Community health workers also had to navigate barriers – such as a lack of education and restricted mobility – that are rooted in gender norms that devalue women and undermine their freedoms" "gender norms undermine the effectiveness of such progressive policies" "there is little evidence that the increased resources resulted in sustained improvements in women's health indicators – which underscores the extent that pernicious gender norms are replicated within the health system." "But institutions such as health systems are embedded in and shaped by the software of health systems [10]: the interactions between people and systems, and within communities and families that are shaped by gender norms. While the concept of 'software' is useful, conceptually it refers to factors that 'float' around the building blocks. We instead utilize the term 'mortar' to indicate that these social norms hold the system together." "Our review suggests that gender equitable health systems that address pernicious gender norms will support both stronger health systems and stronger societies" "Our review suggests that gender equitable health systems that address pernicious gender norms will support both stronger health systems and stronger societies"

Nxumalo, Nonhlanhla. Goudge, Jane. Gilson, Lucy. et al. 2018	Performance management in times of change: experiences of implementing a performance assessment system in a district in South Africa	International journal for equity in health	SVs inform management practice (may conflict with formal procedures)	"The district reportedly made the decision to recruit from within the area as part of the broader provincial imperative (embedded within State policies to address economic and skills inequities) to create opportunities for employment and build skills in the community. This practice is the manifestation of the social and cultural values of the broader South African society where there is the inherent belief that there should be collective benefits of a new democracy." "Culturally-related work values can therefore affect one's interpretation of a performance dimensio" "Ghana's traditional ethos renders a society which places collective values over western-influences of individualism [8, 46]. A similar dynamic was at play in our findings where the district's recruitment processes were fostered by drawing from the community as a means of sharing the benefits of a new democracy, hence empowerment through employment."
Bertscher, Adam. London, Leslie. Orgill, Marsha. 2018	Unpacking policy formulation and industry influence: the case of the draft control of marketing of alcoholic beverages bill in South Africa	Health Policy and Planning	Framing of H pol reflects SVs	"Lastly, perception strategies seek to change the way people think about and frame problems and solutions for health reform, which is linked to the way people view the problem and solution as being consistent or contradicting national symbols, values or identity"
Sabin, J. E. 2018	Rationing Care through Collaboration and Shared Values	Hastings Cent Rep	shared values → HP behaviour & prescribing practices	"having physician incentives built on shared values is the ideal approach to shaping physician behavior"
Malekinejad, Mohsen. Horvath, Hacsi. Snyder, Harry. et al. 2018	The discordance between evidence and health policy in the United States: the science of translational research and the critical role of diverse stakeholders	Health research policy and systems	Pol makers are influenced by social values. SVs impact effectiveness of interventions. SVs influences Pol maker uptake of evidence.	"many competing social values may drive public policy, and better health is only one of them." "any consideration of stakeholder values and preferences must include not only deeply held cultural beliefs, including beliefs about the appropriate role of government, but also other subjective forces such as social stigma." "Interventions shown to be efficacious in well-controlled study conditions may have very different effects in real-world settings or in populations with different cultural values and norms." "Health researchers may immunise themselves by...understanding the culture and values surrounding the problem and policy in question in order to more effectively communicate the scientific evidence to a broader audience outside of the scientific community."

Nguyen, Tuan A. Knight, Rosemary. Mant, Andrea. et al. 2018	Corruption practices in drug prescribing in Vietnam–an analysis based on qualitative interviews	BMC health services research	SVs infludence bh of HS actors.	"For example, the category “rationalization for corruption” was divided into sub-categories of “normalization of corruption” and “self-interest maximization”. Sub-category “normalization of corruption” was further divided into the sub-sub-categories “prevalence of corruption” and “other social norms”." "The pragmatism of mutual support seemed to reinforce the normalizing of corruption. Pharmaceutical company participants said that giving commissions to prescribers was “anormal thing” because “thanks to their [doctors’] contribution, our company can develop.” Therefore, “when the company has profit, our customers [the doctors] have to have profit too.” This logic was also used by doctor participants"
Hanefeld, Johanna. Mayhew, Susannah. Legido-Quigley, Helena. et al. 2018	Towards an understanding of resilience: responding to health systems shocks	Health Policy and Planning	SVs→ how HS responds to shocks. Vs of HCWs and pol makers shape HS structures and processes. SVs → pol priority. Global interventions shape local SVs. SVs → HCW bh. Alignment btwn pol and SVs → pol success.	"people, processes, systems, power relations and values are an integral and mutually dependent parts of the health systems." 'values are fundamental to all health system aspects." "Values have an important role in shaping responses and preparedness but are not often considered." "Values can also be the rationale for deciding to take action or respond in crises." "A focus on inputs or structures (such as theWHO blocks) has insufficient recognition of how health policy development, prioritization and health system structures and processes are shaped by the values, beliefs and preferences of the actors within a health system." "‘Values’ is used here to encompass a range of dimensions, including the political priority given to health during an external shock as well as societal values in which the health system and its workers are embedded, and the personal, professional and societal moral landscapes that play a particularly important role in how difficult decisions are negotiated and compromises reached." 'international humanitarian interventions shape and interact with local values shared by health workers, patients and communities." "Health worker compassion was evident in some European countries when their governments opted to exclude undocumented migrants from the health system: healthcare professionals continued providing services to this group arguing itwas against their ethics and core values as healthcare professionals" "recognizing and aligning policy with ‘values’ underpinning health systems affect whether interventions in each block are succeeding."
Kapiriri, Lydia. Chanda-Kapata, Pascalina. 2018	The quest for a framework for sustainable and institutionalised priority-setting for health research in a low-resource setting: the case of Zambia	Health research policy and systems	SVs → priority setting	"We also highlight the need to contextualise the criteria for ranking priorities in order to reflect local values. The HRPS approaches should be flexible so that they can accommodate any locally prioritised values, which may not be included in the standard approaches."
