# Supplementary material for: Towards an Explanation of the Social Value of Health Systems: An Interpretive Synthesis
Source: Int J Health Policy Manag. 2020 Aug 26;10(7):414–29. doi: 10.34172/ijhpm.2020.159 (PMC9056134; doi:10.34172/ijhpm.2020.159)
Supplement: Supplementary file 2 — Meta-Ethnography. [file ijhpm-10-414-s002.pdf]

Supplementary file 2. Meta-Ethnography

Description & Rationale:

Developed by Noblit and Hare, meta-ethnography is an interpretive approach that allows analysts to synthesise qualitative data, including assumptions, approaches and findings of primary texts[1,2] to elaborate "current understandings and render them more interpretable."[2] Emerging as a response to qualitative synthesis approaches that obscure the richness of the primary data, offering "summations rather than explanations," and therefore making it difficult to draw any explanatory conclusions from the synthesis, meta-ethnography compares data across sources, to enable new, holistic interpretations that are based in, but go beyond that data, and offer a theory that explains the data.[1-3] Insofar as meta-ethnography is generative rather than summative, it is an ideal approach for synthesis that seeks to retain the nuance and complexity of primary data to build new interpretations and explanatory theory.[3]

Given the particular nature of the data to be synthesised—qualitative data from a range of sources offering various conceptualisations of the relationship between health systems and social values—it was necessary to find an approach to data analysis that would allow us to combine underlying assumptions with explicit findings in the same set of primary data. In addition, we sought an approach that would allow us to use the primary data to develop a unified argument about how the health system could possess this productive capacity. Meta-ethnography allows for the combination of underlying assumptions and findings within a single data-set, and, in certain circumstances[i], facilitates the identification of the relationship between data points in terms of a single line of argument, or in this case, explanatory theory.[2,3] Accordingly, we drew on meta-ethnographic analytic tools to determine how the raw extracts were related to one another, translate extracts from primary texts into a common frame and synthesise data to facilitate comparison across extracts, and ultimately use the synthesized data to generate new explanatory theory.

Steps for meta-ethnography:[1,3,4]

1. Getting started—Identify an area of interest worthy of synthesis.

The need for this synthesis is a product of the findings of the original review, which suggested that HS have the capacity to generate social values, but did not suggest an explanation for this capacity.

2. Purposive searching and selecting of relevant studies.

Data selection was conducted iteratively through a qualitative systematic review of social values in HPSR literature reported briefly in supplementary material 3, and more fully in Whyle & Olivier (2020).[5]

3. Reading the studies to identify raw data for the synthesis.

Texts were read in full. Claims about the relationship between health systems and social values extracted verbatim. Apparent underlying assumptions or conceptualisations of this relationship were noted.

4. Determining how the studies are related—either directly comparable, oppositional, or together sustain a line of argument.

Taken together, the data from the primary literature can be interpreted as a single line of argument—suggesting an explanatory theory for the social value of health system. This explanatory theory represents a new interpretation or theory that encompasses and applies across the primary data set.

5. Translating the studies into one another—mapping concepts onto one another to identify similarities and differences.

We mapped the relational claims identified in the primary literature in a single diagram allowing for interpretation based on a common metaphore. This was possible because the relational claims are founded on a common conceptual foundation: that of the health system as a network of interactions.

6. Synthesising the translations by identifying concepts, frameworks or theories that transcend individual texts and produce new interpretations and explanations.

Applying concepts from complex adaptive systems theory, we present an explanation for the capacity of health systems to generate social values as an emergent property of complexity.

7. Expressing the synthesis in a way that is intelligible to the intended audience of the original data set.

We introduce the reader to key concepts from CAS theory, and illustrate the explanatory theory using examples from the original data set.

References

1. Pope C, Mays N, Popay J. *Synthesising qualitative and quantitative health evidence: A guide to methods*: McGraw-Hill Education (UK); 2007.

2. Noblit GW, Hare RD. Meta-Ethnography: Issues in the Synthesis and Replication of Qualitative Research. 1983.
3. Noblit GW, Hare RD. *Meta-ethnography: Synthesizing qualitative studies.* Vol 11: sage; 1988.
4. McCormick J, Rodney P, Varcoe C. Reinterpretations across studies: An approach to meta-analysis. *Qualitative health research.* 2003;13(7):933-944.
5. Whyle E, Olivier J. 2020. Social values and health systems in health policy and systems research: a mixed-method systematic review and evidence map. Health Policy & Planning, czaa038.

---

[i] Noblit and Hare (1988) suggest that presuming a common line of argument across the primary data is appropriate when the primary papers are not opposition, but are also not directly analogous.
