# Supplementary material for: Towards an Explanation of the Social Value of Health Systems: An Interpretive Synthesis
Source: Int J Health Policy Manag. 2020 Aug 26;10(7):414–29. doi: 10.34172/ijhpm.2020.159 (PMC9056134; doi:10.34172/ijhpm.2020.159)
Supplement: Supplementary file 3 — Data Collection and Sampling for the Prior Mixed Methods Review. [file ijhpm-10-414-s003.pdf]

Supplementary file 3. Data Collection and Sampling for the Prior Mixed Methods Review

[Note: This review was published in full as: Whyle E, Olivier J. 2020. Social values and health systems in health policy and systems research: a mixed-method systematic review and evidence map. Health Policy & Planning, czaa038.]

The first phase consisted of a formative scoping review for work referring to 'social values' in health policy processes and health systems. In the second phase, a search strategy for use in the PubMed database was developed on the basis of the scoping review. Two search strings were developed, the first comprising 'social values' and variations thereof, and the second comprising topic terms such as 'health system,' 'policy,' or 'planning' (restricted to title or abstract).

The third phase of data collection consisted of a systematic search of the published outputs of 23 prominent authors in the field of HPSR. The key authors were identified through a meta-analysis of the most commonly recurring authors in a search for 'HPSR OR health policy and systems research' conducted on Scopus. This list was checked against database of materials gathered through the formative scoping review to ensure that the list was sufficiently comprehensive. The final list of key authors included: Abimbola, S; Adam, T; Agyepong, I A; Bennett, S; Bigdeli, M; Boom, G; Daniels, K; El-Jardali, F; George, A; Ghaffar, A; Gilson, L; Hyder, A A; Lehman, U; Marchal, B; Mills, A; Mirzoev, T; Molyneux, S; Peters, D H; Pratt, B; Sheikh, K; Theobold, S; Tran, N; Uzochukwu, B.

For each of the 23 key authors, a Google Scholar search was conducted for articles by that author using terms in the 'social values' search string, and relevant material identified for inclusion through title and abstract screening.

In the fourth phase, a targeted search of key HPSR journals was conducted. The list of key journals developed initially through the scoping review, and amended on the basis of consultation with field experts. Ultimately 11 journals were identified: BMC Global Health; BMC Health Services Research; Health Affairs; Health Policy; Health Policy and Planning; Health Research Policy and Systems; International Journal for Equity in Health; International Journal of Health Policy and Management; Journal of Health Politics, Policy and Law; Medical Anthropology Quarterly; Social Science and Medicine. Each journal was searched through the journal website using the 'social values' search string, and titles and abstracts screened to identify material for inclusion.

Finally, we searched the publications lists of key organisations in the field (again, as identified by the field experts, and by searching within each organisational database for the phrases in the

‘social values’ search string), including the Alliance for Health Policy and Systems Research, World Health Organization, Health Systems Global, and the Collaboration for Health Policy and Systems Analysis in Africa.

In addition to the use of ‘social values’ or a similar phrase, the full text of each paper was screened for eligibility based on two criteria. First, papers were screened to ensure they fell within the bounds of the field of HPSR. As HPSR has notoriously ‘fuzzy boundaries,’[1-3] six guiding principles were applied: addresses system-level issues; utilises a prominent HPSR framework; one or more of the papers’ authors list an institute or department focusing specifically on policy and systems as a primary affiliation; published in a policy or systems-relevant journal; the other work of any of the authors is largely HPSR-focused; and the reference list includes key HPSR texts. In general, papers had to meet more than one of these criteria to be considered relevant. Next, the remaining papers were read in full, and data on the proposed relationship between social values and health systems identified.

The initial search, conducted in September and October of 2018, identified 316 items across all four phases (see Figure). During this process, an additional 97 items were added through preliminary author and citation tracking. After full text screening for relevance 223 items were excluded, and an additional 46 items were included through author and citation tracking.

Many of the papers were analyses of health systems or health policies, or focused on health system reform efforts. However, the largest proportion of papers were about priority-setting. Almost half of the papers were not empirical, including opinion pieces, editorials and commentaries, as well as articles in which methods and findings are not explicitly laid out, but which are presented as based on common knowledge, existing evidence, or personal experience. Of the empirical papers, almost all were qualitative studies of primary or secondary evidence, while a few were mixed methods or quantitative. Overwhelmingly, the included papers were produced in, and present research conducted in high-income countries, with the USA, UK and Canada particularly well represented.[4]

Figure: Flow diagram of study selection procedure and results

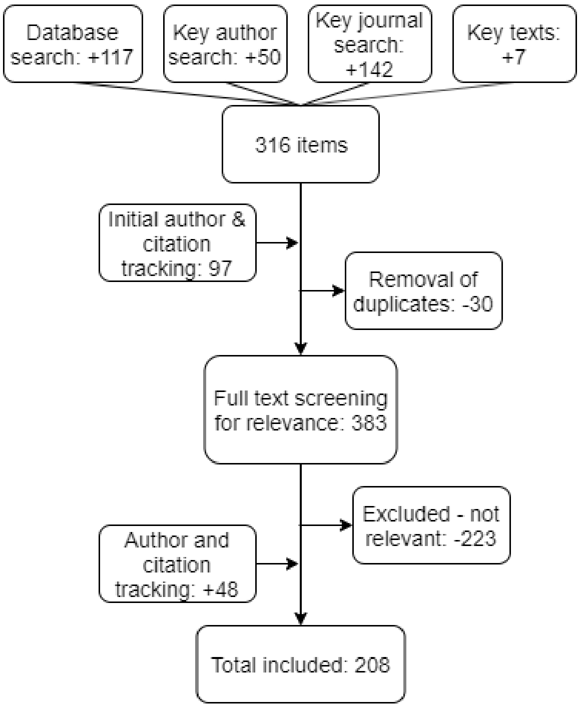

References

1. Gilson L. *Health policy and systems research: A methodology reader*. Geneva: Alliance for Health Policy and System Research: WHO; 2012.

2. Pratt B, Paul A, Hyder AA, Ali J. Ethics of health policy and systems research: a scoping review of the literature. *Health policy and planning.* 2017;32(6):890-910.

3. Hoffman SJ, Røttingen J-A, Bennett S, Lavis JN, Edge JS, Frenk J. *Background paper on conceptual issues related to health systems research to inform a WHO global strategy on health systems research.* Geneva, Switzerland: World Health Organization; 2012.

4. Whyle E, Olivier J. 2020. Social values and health systems in health policy and systems research: a mixed-method systematic review and evidence map. Health Policy & Planning, czaa038.
